# PRAgmatic Clinical Trial Design of Integrative MediCinE (PRACTICE): A Focus Group Series and Systematic Review on Trials of Diabetes and Kidney Disease

**DOI:** 10.3389/fmed.2021.668913

**Published:** 2021-08-27

**Authors:** Kam Wa Chan, Pak Wing Lee, Crystal Pui-sha Leung, Yee Kwan Law, Lucy Gao, Gary Chi-wang Chan, Wai Han Yiu, Tai Pong Lam, Sydney Chi-wai Tang

**Affiliations:** ^1^Department of Medicine, The University of Hong Kong, Hong Kong, China; ^2^Faculty of Epidemiology and Population Health, London School of Hygiene & Tropical Medicine, London, United Kingdom; ^3^Department of Family Medicine and Primary Healthcare, Hong Kong East Cluster, Hospital Authority, Hong Kong, China; ^4^Department of Biochemistry, University of Oxford, Oxford, United Kingdom; ^5^Department of Family Medicine and Primary Care, The University of Hong Kong, Hong Kong, China

**Keywords:** integrative medicine, method, qualitative, pragmatic, clinical trial, systematic review, diabetes, kidney

## Abstract

**Background:** Pragmatic trials inform clinical decision with better generalizability and can bridge different streams of medicine. This study collated the expectations regarding pragmatic trial design of integrative medicine (IM) for diabetes and kidney diseases among patients and physicians. Dissonance between users' perspective and existing pragmatic trial design was identified. The association between risk of bias and pragmatism of study design was assessed.

**Method:** A 10-group semi-structured focus group interview series [21 patients, 14 conventional medicine (ConM) and 15 Chinese medicine (CM) physicians] were purposively sampled from private and public clinics in Hong Kong. Perspectives were qualitatively analyzed by constant comparative method. A systematic search of four databases was performed to identify existing IM pragmatic clinical trials in diabetes or kidney disease. Primary outcomes were the pragmatism, risk of bias, and rationale of the study design. Risk of bias and pragmatism were assessed based on Cochrane risk-of-bias tool and PRECIS-2, respectively. The correlation between risk of bias and pragmatism was assessed by regression models with sensitivity analyses.

**Results:** The subtheme on the motivation to seek IM service was analyzed, covering the perceived limitation of ConM effect, perceived benefits of IM service, and assessment of IM effectiveness. Patients expected IM service to retard disease progression, stabilize concomitant drug dosage, and reduce potential side effects associated with ConM. In the systematic review, 25 studies from six countries were included covering CM, Korean medicine, Ayurvedic medicine, and western herbal medicine. Existing study designs did not include a detailed assessment of concomitant drug change and adverse events. Majority of studies either recruited a non-representative proportion of patients as traditional, complementary, and integrative medicine (TCIM) diagnosis was used as inclusion criteria, or not reflecting the real-world practice of TCIM by completely dropping TCIM diagnosis in the trial design. Consultation follow-up frequency is the least pragmatic domain. Increase in pragmatism did not associate with a higher risk of bias.

**Conclusion:** Existing IM pragmatic trial design does not match the patients' expectation in the analysis of incident concomitant drug change and adverse events. A two-layer design incorporating TCIM diagnosis as a stratification factor maximizes the generalizability of evidence and real-world translation of both ConM and TCIM.

## Existing Evidence

Pragmatic trials better reflect real-world effectiveness of interventions. Integrative medicine (IM) amalgamates multiple streams of medicine with different disease classifications and treatment strategies which require pragmatic assessment. However, existing pragmatic trial design seldom considers users' perspective and there are concerns on whether flexibilities in pragmatic trial design would compromise internal validity.

## Key Contributions to the Literature

1. This is the first focus group series to explore the expected outcomes of patients and physicians regarding pragmatic trial design of IM for diabetes and renal service, involving patients and family medicine, internal medicine, and Chinese medicine (CM) physicians. Unmatched expectation in existing studies was identified through systematic review.
2. Patients expected integrative Chinese-western medicine service to retard disease progression, stabilize concomitant drug dosage, and reduce potential side effects associated with conventional treatment.
3. Existing IM pragmatic trial designs did not include detailed assessment of concomitant drug change and adverse events. Consultation follow-up frequency is the least pragmatic domain in existing IM pragmatic trials.
4. Majority of studies either recruited a non-representative proportion of patients by using traditional, complementary, and integrative medicine (TCIM) diagnosis as inclusion criteria, or not reflecting the real-world practice of TCIM by completely dropping TCIM diagnosis.
5. Increase in pragmatism in study design did not associate with a higher risk of bias from existing evidence.

## Implications

Existing IM pragmatic trial design does not match users' expectation in the analysis of incident concomitant drug changes and adverse events. A two-layer design incorporating TCIM diagnosis as a stratification factor maximizes the generalizability of evidence and real-world translation for both conventional medicine and TCIM.

## Introduction

Pragmatic trials evaluate the effectiveness of interventions in the real-world setting aiming to inform clinical decision and implementation with better generalizability (1, 2). Compared to conventional phase III randomized controlled trials, pragmatic trials often are open-label, have less stringent inclusion/exclusion criteria, involve complex/flexible interventions, compare to usual care, and measure outcomes that are patient-centered (1, 2). Integrative medicine (IM) amalgamates conventional medicine (ConM) and other streams of medicine from a patient-centered and effectiveness-driven approach (3–5).

Traditional, complementary, and integrative medicine (TCIM), including Chinese medicine (CM), naturopathic medicine, mind–body therapies, and other streams of medicine, are often personalized as their theories were developed predominantly from expert consensus and case series (6). Differences in epistemology (for instance, disease classification and treatment strategy) between ConM and TCIM led to controversies in the evaluation of TCIM's effectiveness (7–10). Most clinical trials and meta-analyses were designed to estimate the adjusted or averaged effectiveness of a regimen from a population of patients. However, the likelihood of being responsive toward a regimen of each individual patient with distinctive demographics and phenotypes is often more needed by a physician in the clinical situation (11–13). There are continuous concerns on the conventional evidence-based paradigm building on meta-analyses and randomized controlled trials with limited personalized design (e.g., prespecified subgroup analysis, responder analysis), such as being over-concentrated in population-based assessment (14, 15), over-standardized treatment (15, 16), and lacking personalization (17). This affected the clinical utility of the evidence (18) and was contradicted with many core principles of TCIM. The efficacy-driven approach, which focuses on comparative effectiveness, has been proposed to bridge ConM and TCIM (8, 19–22).

Stakeholder (e.g., patients and physicians) engagement is the foundation of designing pragmatic studies (2, 23). Stakeholder involvement in the study design stage, from the selection of disease condition, drug formulation, and outcome measurement, is increasingly emphasized to enhance the clinical utility and translation of evidence (18, 24). Nevertheless, there are controversies over the pragmatic features (e.g., unblinding of subjects, no placebo control, intervention adjustment) as these flexibilities may enhance generalizability at the expense of internal validity of the evidence (25, 26). The correlation between risk of bias and pragmatism remains unclear.

Diabetes presented in 9.5% of adult population and accounted for 9.9% of all-cause mortality globally (27, 28). The healthcare expenditure on diabetes mounted to US $850 billion worldwide in 2017, representing 11.6% of the total health expenditure (27, 28). Both diabetes and kidney dysfunction are the top 10 conditions attributed to disability-adjusted life-years among population aged over 25 globally (29). In the past decade, CM formulations have been reported to protect against diabetes and chronic kidney disease (CKD) *via* orchestrated mechanisms (30–35). However, less than 2% of diabetic patients have ever used CM for diabetes or CKD in Hong Kong which was substantially lower than the utilization in other disciplines (e.g., 50% for cancer patients) (36). Lack of high-quality and communicable evidence has been suggested as one of the key obstacles in implementing IM (6).

This study aimed to collate and explore the expectations regarding the pragmatic trial design of IM for diabetes among patients and physicians. Subsequently, the existing trial design was systematically assessed to identify the dissonance with users' perspective.

## Methods

### Study Design

A 10-group semi-structured focus group interview series was conducted among patients and physicians with constant comparative method to explore their expectation regarding the IM management of diabetes in general (37). Seven high-level themes were previously identified from the interview series. Two themes regarding the barriers to access and the preferred delivery mode of health services were reported (6). In this study, we report another major theme related to pragmatic trial design. A systematic review was conducted subsequently to contrast existing IM pragmatic trials to the users' perspectives identified from the focus group interviews.

### Focus Group Interview

The focus group interview series was designed to explore the expectations and concerns of the patients and physicians regarding the IM service access and further research. Detail of the interview methods was previously described (6). Briefly, 50 subjects (21 diabetes patients, 14 ConM physicians, and 15 CM physicians) with diverse demographics and experience were purposely sampled from public clinics, private clinics and teaching hospitals in Hong Kong. A series of face-to-face group interviews with three groups of 6–8 patients, three groups of 3–6 ConM physicians, and four groups of 3–4 CM physicians were conducted. Each interview lasted 60–120 min allowing at least 20 min per participant for adequate interaction. CM physicians were sampled to represent TCIM in Hong Kong as CM is the mainstream of TCIM, and integrative Chinese-western medicine is the major form of IM globally including Hong Kong (38).

The interviews were facilitated by a moderator (P.W.L) with relevant experience and conducted in Cantonese (native language of participants). The identity of interviewees and the moderator was blinded before the interview took place. The interviews were built around participants' consultation experience, concerns and expectations based on a semi-structured interview guide (6). The process of recruitment, interview and analysis were iterative until data saturation during the last round of interview (patient and ConM: third round, CM: fourth round). Interview content was analyzed by constant comparative method (37). Maximum codes on main themes and subthemes were first generated independently by two bilingual investigators (K.W.C., P.W.L.) for initial open coding with revisit to check for emerging ideas. The concepts and theories were refined, and the association of the coding was explored to form axial coding. Final core coding was formed after data saturation and was applied to index the whole dataset. Charted result was translated by a bilingual investigator (K.W.C.) when used as illustrative quotations. Data were processed with the support of simple software (Microsoft Word and Excel) for convenient access.

### Systematic Review

#### Search

We sought to assess the pragmatism, risk of bias, and rationale of study design of the existing pragmatic trials of diabetes and kidney disease using IM as intervention. The search strategy (Supplementary File 1) was formulated to include all IM pragmatic clinical trials and trial protocols that recruit patients with diabetes or kidney diseases published until 24 August 2020. IM included any intervention that is not conventionally used in clinical practice, for instance, herbal medicine, acupuncture, and massage. Four databases were searched including Cochrane, Medline, Embase and PubMed. Reference lists were also searched. A clinical epidemiologist (K.W.C.) led the search and data processing. Endnote X9 was used to aid the review process. Protocol registration: CRD4D2021231288.

#### Screening

After removing duplicated studies, screening started with title and abstract followed by full text before data extraction. All articles were dually screened, assessed, and extracted (Y.K.L, L.G.) independently with a standardized form. All disagreements were resolved by discussion and determined by K.W.C. if consensus could not be reached. There was no language restriction. All observational and qualitative studies were excluded. Studies that used health services or supplements as intervention were excluded.

#### Quality Assessment and Data Extraction

The co-primary outcomes were the pragmatism, risk of bias, and rationale of the study design. Pragmatism of the trials was assessed based on the PRECIS-2 tool (39) on study population, recruitment setting, intervention delivery, and outcome assessment. Risk of bias in randomization, allocation concealment, blinding, incomplete outcome data, and selective reporting was assessed based on the Cochrane risk-of-bias tool (40). The rationale of study design in target population, intervention, comparator, and outcome assessment were identified from the study.

#### Statistical Analysis

The correlation between risk of bias and pragmatism was assessed by univariable and backward multivariable regression analysis adjusting publication year and sample size. For the quantified assessment of the overall risk of bias of each study, the scores of low, unknown, and high risk were given 0, 1, and 2 points. Lower total score represents low risk of bias in the reported study design. For pragmatism, each domain scored 1 for being least pragmatic and 5 for being most pragmatic, respectively, according to the guideline from the PRECIS-2 tool. For domains that were not assessable, the score was replaced by 3 (midpoint). As there is no consensus on the statistical handling of undetermined domains, sensitivity analysis was conducted to replace undetermined domains by 1 and 5 to test the robustness of results. STATA 15.1 was used for analysis.

## Results

### Focus Group Interviews

**Figure 1:**
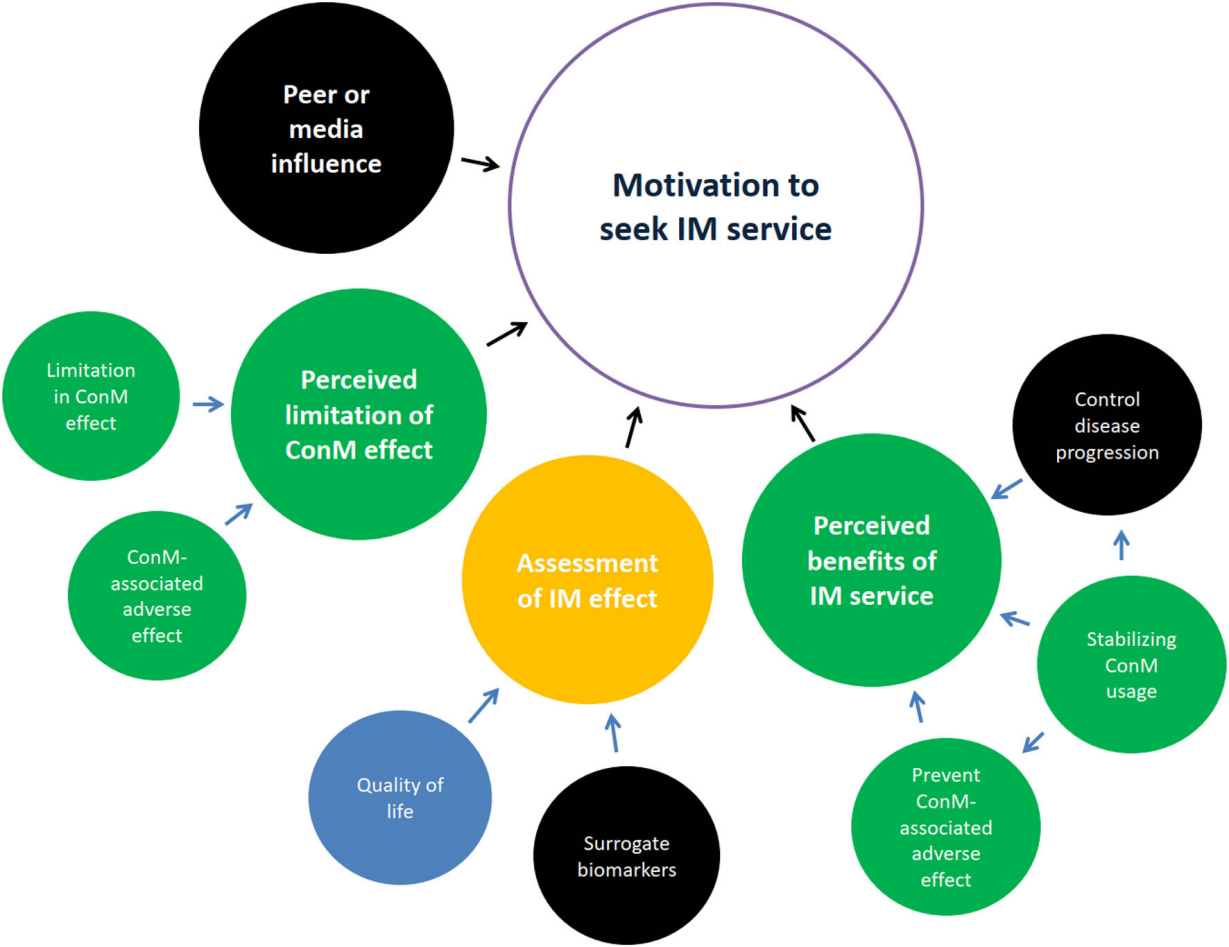
Motivation to seek integrative medicine (IM) service. Themes generally agreed upon by patients in yellow, by Chinese medicine (CM) physicians in blue, by conventional medicine (ConM) physicians in red, by both patient and CM physicians in green, by both patient and ConM clinician in orange, by all parties in black. Control of disease progression was the common perceived benefit of IM. Stabilizing ConM usage was emphasized by patients and acknowledged by CM physicians. Surrogate biomarkers were mutually accepted among patients and physicians. Importance on quality of life divided between patients and CM physicians.

Majority of patients had poor glycemic control (71.4%), with stage 2–4 CKD (95.2%) and albuminuria (90.5%); 4.8% of patients reached end-stage kidney failure, 57.1% (*n* = 8/14) of ConM physicians specialized in internal medicine, 42.9% (*n* = 6/14) of ConM physicians specialized in family medicine or practiced as general practitioners, 42.9% (*n* = 6/14) of ConM physicians received CM education, and all (*n* = 15) CM physicians received substantial credit bearing ConM education from their undergraduate study. Seven high-level themes, namely, barriers toward IM service, motivation to seek CM service, background knowledge on diabetes, experience of CM service, preferred model of integrative service delivery, and evidence of IM and CM hospital, were previously identified leading to 25 subthemes (6). Data on a high-level theme: motivation to seek IM service is related to the clinical trial design and reported in this study (Figure 1). Quotes are summarized in Table 1.

**Table 1:** Subthemes and illustrative quotations of focus group interview.

**Motivation to seek integrative medicine service**	**Source**	**Illustrative quotations**
**Subtheme: perceived limitation of ConM effect**		
Limitation in ConM efficacy	Patient	“I have never got better with ConM. My kidney function is falling down…They always say that I am not going to recover. I can only wait for dialysis or transplantation.” (Patient 21)
	ConM physician	“If I have done whatever I could do and the kidney function is still deteriorating, and there is a (CM) formulation that the patient may try, then the patient may want to try…that is if I can do nothing, you may try, but do no harm.” (ConM physician 8)
ConM-associated adverse effect	Patient	“ConM cannot control my blood glucose. It fluctuated a lot. I tried to have CM for 1 year and the blood glucose was stabilized.” (Patient 6)
	Patient	“They said there is a drug (ACEI/ARB) which can help my kidney but I could not take it as my (serum) potassium elevated. High (serum) potassium is even worse as it affects the heart.” (Patient 18)
	CM physician	“Some patients were having poor liver function or hypersensitivity toward ConM and they came…they thought CM is natural and have a lower risk.” (of toxicity).” (CM physician 1)
**Subtheme: perceived benefits of IM service**		
Better control of disease progression	Patient	“Kidney is the most important. We need dialysis once it deteriorated.” (Patient 8)
	ConM physician	“It would be the best if CM can control diabetes and slower the progression of DKD as there is a group of patients deteriorated quite fast. Retarding the renal progression would be an important achievement.” (WM physician 13)
	CM physician	“Patients that are highly educated and younger focused more on (laboratory) investigations. Older patients focused more on quality of life and wished CM can help.” (CM physician 4)
Stabilizing ConM usage, preventing the associated adverse effects	Patient	“(I would like to have) less ConM intake and consultation.” (Patient 4, 5, 7)
	CM physician	“Majority of patients were reluctant to take ConM as they believed they could not stop (taking ConM) once started. They were willing to try alternatives including CM.” (CM physician 2)
**Subtheme: assessment of IM effectiveness**		
Surrogate biomarkers	Patient	“Data (investigation) is more objective as it can be measured.” (Patient 2)“Kidney index (serum creatinine), urine protein.” (Patient 17)
	ConM physician	“GFR, creatinine, urine protein, those routine measures.” (ConM physician 6)“The kidney function may get worse even you treat the ‘blood and qi'. There are some mismatch on the outcomes… you (CM) have to match ours (outcome measures) …There can be many outcomes but we have to be in the same direction… Those investigations (GFR, UACR, LFT) are a must for us, ConM clinicians. It would be hard for us to accept that we have to depend on other outcome measures just because we work with a CM physician.” (ConM physician 9)
	CM physician	“DKD is (a condition) defined by ConM. We have to refer to ConM (investigations) for treatment. If the disease is classified by CM, then it should be referring to CM (outcome measures).” (CM physician 2)
Quality of life	ConM physician	“I always think that CM is totally different (when compared to ConM) from principles to treatment strategy. I do not understand what they measure and how they formulate treatment. They may work and I am not sure if (lab) investigation is a must for them.” (ConM physician 13)“I am not sure if CM can go further into molecular level…if you can explain the pharmacology of every drug based on statistics, it would be a huge advancement.” (ConM physician 2)
	CM physician	“I had a patient with long diabetes history and had good control on investigation markers. However, he has got symptoms of *spleen and kidney deficiency*. I believe his life expectancy and quality of life will get better with CM. It cannot be shown without CM assessment.” (CM physician 11)“For elderly, the markers are not important except being rapidly deteriorating. Younger patients are more concerned about markers…that is, it (the outcome measurement) has to be personalized.” (CM physician 15)

*Perspectives of patients, conventional medicine (ConM) physicians and Chinese medicine (CM) physicians were compared*.

*Themes generally agreed upon by patients in yellow, by Chinese medicine (CM) physicians in blue, and by conventional medicine (ConM) physicians in red*.

### Main Theme: Motivation to Seek IM Service

Four subthemes related to the motivation of seeking IM service were identified, namely, (1) perceived limitation of ConM effect, (2) peer or media influence, (3) perceived benefits of IM service, and (4) assessment of IM effectiveness. Subthemes 1, 3, and 4 are relevant to study design and summarized below.

#### Subtheme: Perceived Limitation of ConM Effect

Majority of patients considered IM as they believed the effect of ConM was limited and was concerned about the adverse effects after receiving ConM.

##### Limitation in ConM Efficacy

Most patients believed that diabetes and diabetic kidney disease (DKD) are irreversible, which was reflected by the limitation of the current regimens (41–44). This prompted patients to explore alternatives for more options to control disease progression. Physicians from both ConM and CM acknowledged that patients generally prefer IM treatment. Majority of patients approached IM when they experienced disease progression, for example, poor blood glucose control, or developed complications including DKD.

##### ConM-Associated Adverse Effect

Patients mentioned their experience in developing adverse effects that perceived to be ConM-associated. These included hypoglycemia, hyperkalemia, diarrhea, fluctuating blood glucose, and fatigue. Majority of patients believed that CM has less adverse effects when compared to ConM. A similar observation was suggested by CM physicians.

#### Subtheme: Perceived Benefits of IM Service

There are several benefits that patients believed IM can offer, including better control of disease progression, prevention of ConM-associated side effect, and stabilizing ConM usage.

##### Better Control of Disease Progression

Patients sought to have better control of disease progression, for instance, reducing the risk of complications and increasing life expectancy when they consider IM. DKD was highlighted as a major concern as patients were reluctant to receive dialysis. Some CM physicians suggested that patients of different age groups had different treatment targets. Elder patients emphasized more on symptomatic improvement and quality of life, while younger patients focused on laboratory investigations. A few CM physicians suggested that CM emphasizes holistic improvement including both quality of life and biomarkers.

Although patients expressed subjective unwell feeling after receiving ConM, symptomatic improvement did not emerged as a major expectation from patients. CM physicians, however, believed that improving quality of life would be a major concern among patients and an advantage of CM. ConM physicians suggested control of renal function deterioration as an important milestone of complication management; however, they emphasized that more evidence is needed to demonstrate such effect of CM.

##### Stabilizing ConM Usage and Preventing the Associated Adverse Effects

Reducing ConM dependence was one of the common expectations of patients. Some CM physicians reported similar requests encountered in their clinical practice. This is likely because patients linked the use of ConM with disease progression and adverse effects. Minority of patients expect CM to reduce the adverse effects of ConM. Some CM physicians suggested that they have managed ConM-associated adverse events.

#### Subtheme: Assessment of IM Effectiveness

Patients generally focused on objective conventional biomarkers measured by laboratory investigations for the monitoring of treatment effect, which was supported by the ConM clinicians. Some CM physicians also believed that objective markers were important for their self-evaluation of treatment effect, as DKD is a ConM-defined condition. They also expected the patients would evaluate their treatment based on laboratory investigation results.

Substantially diverted opinion was noted among CM physicians, suggesting current biomarkers should not be the only outcome assessment. They believe CM manages patients' general condition simultaneously while treating DKD. DKD-related biomarkers were limited to only reflect a certain aspect of patients' overall condition. They suggest the concurrent use of CM-related outcome measures, which is phenome-based (e.g., change in symptoms, tongue color and pulse form).

Some ConM physicians acknowledged the difference in the epistemology between CM and ConM and suggested that CM may require different outcome measures. Nevertheless, ConM physicians generally believed that it would be an advantage if the effect of CM can be demonstrated with study designs conventionally used in ConM. There was also a suggestion to personalize the assessment of effect based on the patients' preference which is related to their demographics.

### Systematic Review

**Figure 2:**
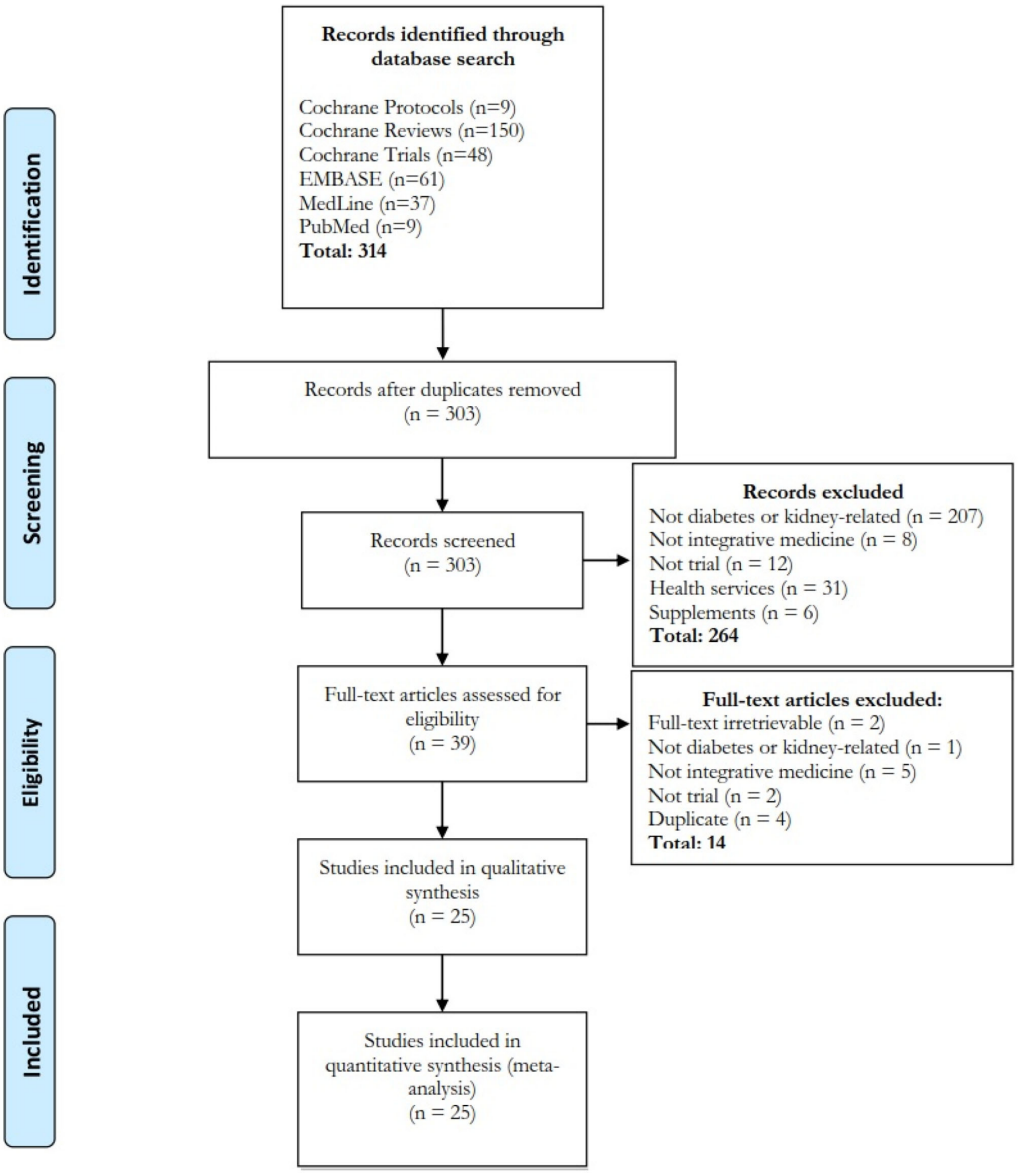
Flow diagram of systematic review. Four databases and search engines were searched and 25 papers were included. Interventions included acupuncture/acupressure (*n* = 7), herbal products (*n* = 14), massage-related (*n* = 2), qigong (*n* = 1) and combined acupuncture-herbal (*n* = 1) treatment. The treatment was formulated according to Chinese (*n* = 20), Kampo (*n* = 2), Korean (*n* = 1), Ayurvedic (*n* = 1), and western herbal (*n* = 1) medicine.

Our search identified 303 studies from four databases after removing duplicated studies (Figure 2; 264 studies were excluded by title and abstract screening and 14 studies were excluded (Supplementary File 2S) after full-text screening. A total of 25 trials were included for analysis.

#### Characteristics of Included Trials

Geographically, 18 (72%), 2 (8%), 2 (8%), 1 (4%), 1 (4%), and 1 (4%) studies were conducted in China, Japan, United Kingdom, United States, Korea, and Sweden, respectively (Table 2). Target population included prediabetic (*n* = 2), diabetic (*n* = 15), glomerulonephritis (*n* = 1), chronic kidney disease (*n* = 2) and hemodialysis (*n* = 5) patients. Complication of diabetes included kidney (*n* = 4), neuropathy (*n* = 4) and arterial disease (*n* = 1). Fifteen were completed trials and 10 were trial protocols.

**Table 2:** Characteristics of included studies.

**First Author**	**Country/year**	**Title**	**Key inclusion/exclusion criteria**	**Size**	**Setting**	**Intervention**	**Frequency**	**Attrition**	**Period**	**Control**	**Primary outcomes**
S. Ono	Japan/2015	Efficacy and Cost effectiveness of the acupuncture treatment using a new skin stimulus tool called m-test which is a measure based on symptoms accompanied with body movements: a pragmatic RCT targeting hemodialysis patients	Hemodialysis patients	47	Outpatient hemo-dialysis facilities	**Acupuncture**	Once weekly. Unknown follow-up frequency	8/47 (17.0%)	8 weeks	Standard care control	20 symptoms evaluated by visual analog scale, quality of life (EQ-5D), cost-effectiveness (ICER)
K. Watanabe	Japan/2016	Long-term effects of goshajinkigan in prevention of diabetic complications: a randomized open-labeled clinical trial	T2DM patients aged 40–75 years with HbA1c over 6.5%	149	Nine clinical centers	**Oral Kampo medicine** (Goshajinkigan extract) preprandially (Rehmanniae radix, Achyranthis radix, Corni fructus, Dioscoreae rhizoma, Hoelen, Plantaginis semen, Alismatis rhizoma, Moutan cortex, Cinnamomi cortex and Aconiti radix)	Three times daily. Unknown follow-up frequency	33/149 (22.1%)	28 months	Standard care control	Incident nonfatal myocardial infarction or nonfatal stroke or stage progression of diabetic nephropathy/retinopathy
C. Elder	USA/2006	Randomized trial of a whole-system ayurvedic protocol for type 2 diabetes	Newly diagnosed T2DM patients aged 21–80 years	60	Kaiser Permanente Center for Health Research Clinic	**(1) Oral Ayurveda herbs**: Phylanthus niruri, Arjuna myrobalan, Eniconstema littlorale, Aegle marmelos, Azadirachta indica, Momordica charantia, blackberry; (2) **transcendental meditation; diet** (fresh cooked vegetables, small legumes, dry light whole grains, and lunch as the main meal); (3) **daily routine and exercise**	Daily. Unknown follow-up frequency	6/60 (10%)	6 months	Standard care control	Glycemic control (HbA1c and fasting glucose levels)
J. Gan	China/2019	Yinang formulation vs. placebo granules as a treatment for chronic kidney disease stages III-IV in patients with autosomal dominant polycystic kidney disease: Study protocol for a double-blind placebo-controlled randomized clinical trial	ADPKD patients aged 18–75 years with Chinese medicine diagnosis of the *spleen, kidney deficiency, and blood stasis* syndrome	72	Outpatient clinics of three university affiliated hospitals	**Oral Chinese medicine** formulation (*Yinang* formulation composed of 17 herbs) twice daily, 1h after breakfast and dinner	Twice daily. Monthly follow-up	N/A	24 weeks	Placebo	Estimated glomerular filtration rate
J. Huo	China/2018	Stationary Treatment Compared with Individualized Chinese Medicine for Type 2 Diabetes Patients with Microvascular Complications: Study Protocol for a Randomized Controlled Trial	T2DM patients aged 18–75 years with Chiense medicine diagnosis of *qi-yin* deficiency and *blood stasis* syndrome and diabetic retinopathy, diabetic kidney disease or diabetic neuropathy	432	Inpatient treatment in 8 Hospitals	Protocoled individualized **Chinese medicine**	3 times daily. Unknown follow-up frequency	N/A	24 weeks	Chinese medicine pill (Qiming granule)	Diabetic retinopathy: changes in retina hemorrhage, retinal exudate, macular thickness, BCVA; diabetic kidney disease: changes in albumin-to-creatinine ratio, serum creatinine and estimated glomerular filtration rate; diabetic peripheral neuropathy: changes in electromyography, TCSS, VAS
D. Jin	China/2019	Chinese herbal medicine Tangshen Formula treatment for type 2 diabetic kidney disease in the early stage: Study protocol for a randomized controlled trial	T2DM patients with microalbuminuria	632	13 Hospitals	**Chinese medicine** formulation (Tangshen Formula)	Twice daily. Monthly follow-up	N/A	24 weeks	Placebo	Urinary albumin-to-creatinine ratio
D. Jin	China/2017	Chinese herbal medicine TangBi Formula treatment of patients with type 2 diabetic distal symmetric polyneuropathy disease: Study protocol for a randomized controlled trial	T2DM patients with polyneuropathy aged 30–70 years	188	Six Hospital clinical centers	**Chinese medicine** formulation (TangBi Formula) two times per day	Twice daily. Monthly follow-up	N/A	24 weeks	Placebo	Changes in clinical signs and symptoms. Changes in Michigan Diabetic Neuropathy Score
Z. Qi	China/2018	Acupuncture combined with hydrotherapy in diabetes patients with mild lower-extremity arterial disease: A prospective, randomized, nonblinded clinical study	Diabetes patients with lower-extremity artery disease	126	Hebei Chronic Disease Rehabilitation Center	**Acupuncture** and low-radon hot spring **thermal hydrotherapy**.	Once every 2 days. Monthly follow-up	5/126 (4.0%)	15 weeks	Standard care control	(1) symptomatic lower-extremity arterial disease assessment, (2) laboratory physical status, and (3) self-report quality of life measures
A. F. Walker	UK/2006	Hypotensive effects of hawthorn for patients with diabetes taking prescription drugs: A randomized controlled trial	T2DM patients with hypertension	79	Outpatient clinics at The University of Reading	Hawthorn (**French herb**) extract 1,200 mg	Twice daily. Monthly follow-up	14/79 (17.7%)	16 weeks	Placebo	Diastolic blood pressure
M. Wang	China/2018	Effects of traditional Chinese herbal medicine in patients with diabetic kidney disease: Study protocol for a randomized controlled trial	Diabetic patients aged 25–75 years with estimated glomerular filtration not <30 ml/min/1.73m2 and (1) albuminuria, (2) diabetic retinopathy, or (3) confirmed biopsy	266	6 Hospitals	**Chinese medicine** formulation according to Chronic kidney stage	Twice daily. Follow-up at baseline, 4, 12, 24 weeks	N/A	24 weeks	Standard care control	Urinary excretion rate, 24-h urine protein and estimated glomerular filtration rate
X. Xie	China/2019	Effect of Gua Sha therapy on patients with diabetic peripheral neuropathy: A randomized controlled trial	Diabetic patients with clinical diagnosis of diabetic peripheral neuropathy aged 18-80 years	113	Not available	Gua Sha (**Chinese medicine physiotherapy**)	Once weekly. Weekly follow-up	6/119 (5.0%)	12 weeks	Standard care control	Validated scale and physical measurement for clinical neuropathy (TCSS, VPT, ABI) and fasting blood glucose
K. W. Chan	China/2016	Semi-individualized Chinese medicine treatment as an adjuvant management for diabetic nephropathy: a pilot add-on, randomized, controlled, multicenter, open-label pragmatic clinical trial	Diabetic kidney disease patients with chronic kidney disease stage 2–3 and macroalbuminuria aged 35 to 80 years	148	8 outpatient clinics	**Chinese medicine** formulations according to symptom-based diagnosis of Chinese medicine practice	Twice daily. Monthly follow-up	N/A	48 weeks	Standard care control	Estimated glomerular filtration rate and urine albumin-to-creatinine ratio
Y. Gao	China/2013	Clinical research of traditional Chinese medical intervention on impaired glucose tolerance	Impaired glucose tolerance patients aged 25–75 years	510	12 clinical centers	**Chinese medicine** formulation (Tangzhiping granules)	Twice daily. Unknown follow-up frequency	52/510 (10.2%)	3 years	Standard care control	Annual conversion rate to T2DM
A. P. Garrow	UK/2014	Role of acupuncture in the management of diabetic painful neuropathy (DPN): a pilot RCT	Diabetic patients with pain neuropathy aged 18–80 years	59	One local district general hospital	**Acupuncture** with five standard acupoints	Once weekly. Weekly follow-up	14/59 (23.7%)	10 weeks	Sham acupuncture	Neuropathic pain by Leeds Assessment of Neuropathic Symptoms and Signs
J. Kou	China/2014	Efficacy and safety of Shenyankangfu tablets for primary glomerulonephritis: study protocol for a randomized controlled trial	(1) diagnosis of primary glomerulonephritis, (2) aged 18–70 years, (3) estimated glomerular filtration rate over 45 mL/min/1.73 m2, (4) 24-h proteinuria level of 0.5–3.0 g, (5) traditional Chinese medicine syndrome conforming to Qi-Yin deficiency	720	Renal outpatient and inpatient departments of a hospital	**Chinese medicine** formulation (Shenyankangfu)	3 days daily. Unknown follow-up frequency	N/A	48 weeks	Placebo and losartan matching shape, size, taste, weight, and color	24-h proteinuria level
S. Lee	Korea/2013	Electroacupuncture to treat painful diabetic neuropathy: study protocol for a three-armed, randomized, controlled pilot trial	Diabetic patients aged 18–75 with painful diabetic neuropathy	45	Outpatient clinic of a university hospital	**Acupuncture** with 12 standard points	Twice weekly. Follow-up twice per week	N/A	8 weeks	Sham acupuncture and usual care group	11-point pain intensity numerical rating scale
W. Mao	China/2015	Rationale and design of the Helping Ease Renal failure with Bupi Yishen compared with the Angiotensin II Antagonist Losartan (HERBAAL) trial: a randomized controlled trial in non-diabetes stage 4 chronic kidney disease	Stage 4 non-diabetic chronic kidney disease patients aged 18–80 years with Chinese medicine diagnosis of *spleen and kidney qi deficiency*	554	16 hospital centers	**Chinese medicine** formulation (Bupiyishen formula)	Three times daily. Unknown follow-up frequency	N/A	12 months	Losartan (standard care)	Estimated glomerular filtration rate
X. Sun	China/2015	The cost-effectiveness analysis of JinQi Jiangtang tablets for the treatment on prediabetes: a randomized, double-blind, placebo-controlled, multicenter design	Prediabetic patients aged 30–70	362	Five hospitals	**Chinese medicine** formulation (JinQi Jiang Tang)	Twice daily. Monthly follow-up	Unknown	12 months	Placebo	Incidence of T2DM
P. A. E. Wandell	Sweden/2013	Effects of tactile massage on metabolic biomarkers in patients with type 2 diabetes	Swedish T2DM patients aged 35–75 years	79	Four primary healthcare centers	**Tactile massage**	Unknown	26/79 (32.9%)	10 weeks	Relaxation exercise	Blood glucose related biomarkers
H. Wang	China/2013	The key role of Shenyan Kangfu tablets, a Chinese patent medicine for diabetic nephropathy: study protocol for a randomized, double-blind and placebo-controlled clinical trial	Diabetic kidney disease patients with diabetic nephropathy stage 3–4 diagnosed with *qi-yin deficiency*	80	Five hospitals	**Chinese medicine** formulation (Shenyan Kangfu tablets)	3 times daily. Follow-up at baseline, 2, 4, 8, 12, 16 weeks	N/A	16 weeks	Placebo	Composite of 24-h urinary protein levels and urinary albumin excretion rate
C. F. Liu	China/2008	Effect of auricular pellet acupressure on antioxidative systems in high-risk diabetes mellitus	High risk diabetes patients	69	Unknown	**Auricular acupressure** (3 points)	Three times daily. Unknown follow-up frequency	Unknown	20 days	Standard care control	Serum superoxide dismutase level
M. Y. Tsai	China/2018	Treatment of intradialytic hypotension with an herbal acupoint therapy in hemodialysis patients: A randomized pilot study	Symptomatic hemodialysis patients aged 20–75 years	32	One academic dialysis center	**Herbal stimulation on acupoint**	Three times weekly. Follow-up three times weekly	5/32 (15.6%)	4 weeks	Placebo	Blood pressure, symptoms, dialysis target
S. L. Tsay	China/2004	Acupressure and fatigue in patients with end-stage renal disease-a randomized controlled trial	Hemodialysis patients aged 18 or above presented with fatigue	106	Four Dialysis centers in major hospitals in Taipei	**Acupressure** (4 points)	3 times weekly. Follow-up 3 times weekly	Unknown	4 weeks	Sham acupuncture	Piper Fatigue Scale, visual analog scale for fatigue, Pittsburgh Sleep Quality Index, Beck Depression Inventory
S. L. Tsay	China/2003	Acupressure and quality of sleep in patients with end-stage renal disease–a randomized controlled trial	Hemodialysis patients with sleep complain aged 18–65 years	98	4 Dialysis centers in major hospitals in Taipei	**Acupressure** (3 points)	3 times weekly. Follow-up 3 times weekly	Unknown	4 weeks	Sham acupuncture on non-acupoints 1 cm away from meridian	Quality of sleep measured by Pittsburgh sleep quality index (PSQI) and sleep log
C. Y. Wu	China/2014	Effect of qigong training on fatigue in haemodialysis patients: A non-randomized controlled trial	Hemodialysis patients aged 18 or above	172	Outpatient dialysis units of a medical center	**Qigong**	Daily. Follow-up three times weekly	6/172 (3.5%)	24 weeks	Standard care control	Fatigue measured by validated Haemodialysis Patients Fatigue Scale

The IM interventions involved included acupuncture/acupressure (*n* = 7), herbal products (*n* = 14), massage-related (*n* = 2), qigong (*n* = 1), and combined acupuncture-herbal (*n* = 1) treatment. The treatment was formulated according to Chinese (*n* = 20), Kampo (*n* = 2), Korean (*n* = 1), Ayurvedic (*n* = 1), and western herbal (*n* = 1) medicine. The median sample size was 113 (IQR: 72–266), and the median treatment duration was 24 weeks (IQR: 10–26). The frequency of treatment ranged from once to three times daily for oral medication and once to three times weekly for acupuncture, respectively. Majority of studies required monthly consultation follow-up for oral medication and three times weekly for acupuncture. Twenty-four studies (96%) either recruited a proportion of patients according to TCIM-specific diagnosis or completely dropped TCIM diagnosis in study design. Five studies included TCIM-specific symptom-based diagnostic criteria in the inclusion/exclusion criteria of study population.

All DKD- related studies used urine albumin/protein and/or estimated glomerular filtration rate (GFR) as primary outcomes. All CKD-related studies assessed estimated GFR as the primary outcome. For hemodialysis-related studies, majority (4/5) assessed quality of life or symptom as primary outcomes. All studies described adverse events narratively. No studies measured the change of concurrent medication as primary or secondary outcomes. Nine, 12, two and two studies used standard care, placebo or sham acupuncture, both standard care and placebo, and other active intervention (e.g., other TCIM medication, active exercise) as comparators, respectively.

#### Risk of Bias, Pragmatism and the Association

**Figure 3:**
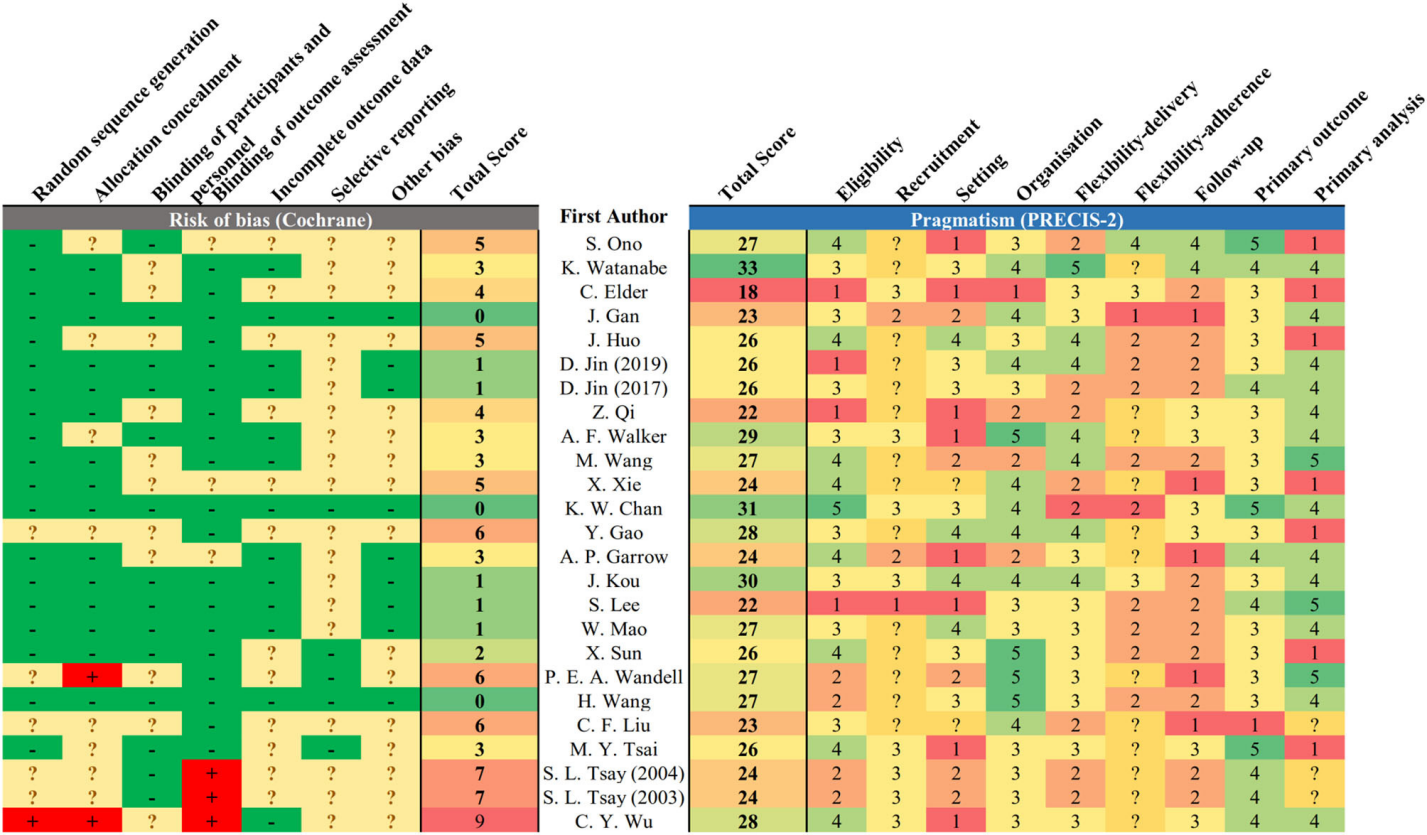
Risk of bias and pragmatism of included studies. The risk of bias was assessed based on the Cochrane risk-of-bias tool, and pragmatism was assessed based on PRECIS-2. Lower total score represents low risk of bias in reported study design. A domain scored 1 or 5 for being least or most pragmatic, respectively, according to the PRECIS-2 tool. Undetermined domain was replaced by 3 (midpoint).

Majority (22/25) of studies reported unclearly in at least one domain of potential bias (Figure 3). Twelve studies had unclear description on handling of attrition that led to undetermined bias on completeness of outcome measurement. Four studies were with high risk of bias in at least one domain. The main source of high-risk bias was from the blinding of outcome assessment (*n* = 3) and allocation concealment (*n* = 2).

**Figure 4:**
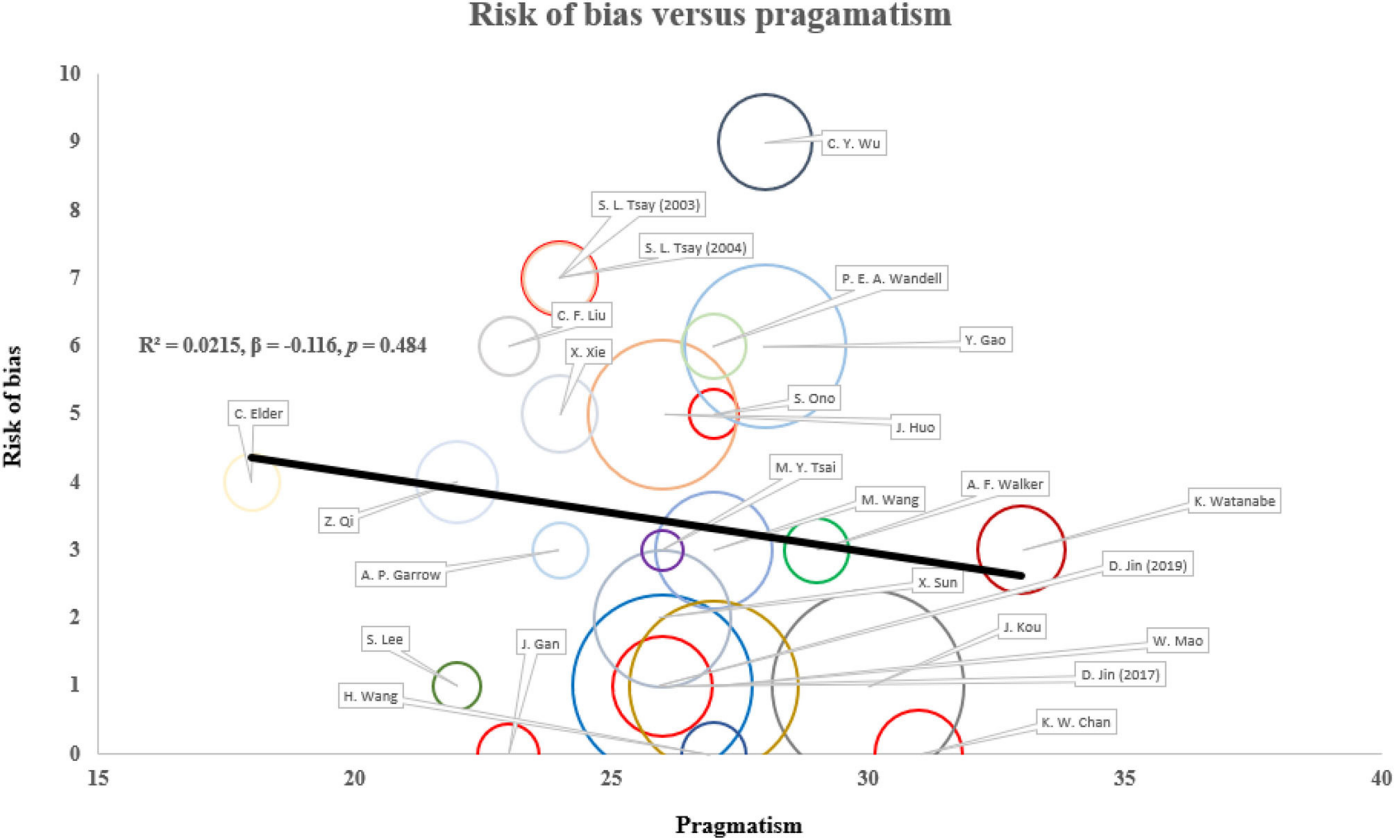
Correlation between risk of bias and pragmatism in existing study designs. The risk of bias and pragmatism was assessed according to the Cochrane risk-of-bias tool and PRECIS-2 tool. Higher score corresponds to higher risk of bias and more pragmatism. Sample size is presented as the size of circle. There is no statistically significant correlation between risk of bias and pragmatism in both unadjusted and adjusted (publication year and sample size) models. Result is robust in sensitivity analyses replacing undetermined domains with extreme values.

In terms of pragmatism, the eligibility and outcome measurement of most trials were close to the target population with limited exclusion criteria (Figure 3). The outcome measurement was mostly relevant to the target population with clinical significance, for instance, the measurement of estimated GFR among DKD and quality of life among dialysis patients. The setting of trials was less pragmatic as most trials require additional expertise to execute on top of existing infrastructure. The follow-up duration was also less practical as the interventions require substantially more frequent service attendance. The reporting on recruitment strategy and adherence control was not clear to assess the degree of pragmatism. There is no observed positive correlation between the risk of bias and pragmatism of the included studies (*R*^2^ = 0.0215, ß = −0.116, *p* = 0.484) (Figure 4). Result was comparable in sensitivity analysis with imputation on undetermined domains in pragmatism (Supplementary File 3S). Replacing undetermined domains in the assessment of pragmatism with lowest value resulted in a negative correlation (*R*^2^ = 0.176, ß = −0.277, *p* = 0.037). Replacement with highest value did not result in significant correlation (*R*^2^ = 0.035, ß = 0.129, *p* = 0.374). The rationale of study design parameters was uncommonly reported. One study used estimated GFR as primary outcome based on conventional practice of other studies. No study included/referred to stakeholder analysis in justifying the study design.

## Discussion

Patients expected IM service to retard disease progression, stabilize concomitant drug (referring to any medications given to the patients except the investigational article) dosage and reduce potential side-effect associated with conventional treatment where existing study designs did not include detailed assessment. Consultation follow-up frequency is the least pragmatic domain in existing studies. Increase in pragmatism in study design did not associate with higher risk of bias.

### Outcome Measures on the Change of Concomitant Drug and Adverse Events

From the focus group interviews, patients expected IM service could retard disease progression, stabilize the use of concomitant drugs, and lower the risk of having adverse events associated with conventional treatment. Surrogate biomarkers were mutually accepted among patients and physicians. Most reviewed pragmatic DKD studies used GFR and urine albumin/protein to measure the change of renal function which addressed both patients' and physicians' preference (6).

Nevertheless, no study in the review reported the change of concomitant regimen as primary or secondary outcomes. Pragmatic trials often involve open-label design to better replicate real-world application. The potential bias in delivering intervention due to unblinding could be adjusted or assessed by mediation analysis on the dynamic change of concomitant regimens. Besides, as clinicians often adjust concomitant drugs to achieve or maintain targets of disease control (e.g., lowering hemoglobin A1c to below 7.0% or lowering systolic blood pressure to below 130 mmHg) in chronic conditions, the change in concomitant drugs could better reflect the disease progression than that of biochemical parameters, which is well-noted by patients in the focus group interviews. While most existing studies included analysis of adverse events, the data collection and assessment methods were unclear, and the reporting was often limited to narrative analyses. Further pragmatic studies should include the change of concomitant regimen as outcome measures and consider performing more systematic and in-depth quantitative analyses (e.g., survival analysis) on the incidence of adverse events.

### Better Adherence by Reducing Intervention and Consultation Follow-Up Frequency

Among the existing studies, the frequency of add-on oral TCIM medication intake was often three times daily. Since the TCIM-ConM drug interaction is a common concern among ConM physicians, add-on oral TCIM medication is commonly taken separately with ConM (6). Therefore, existing IM study protocols require patients to take medication five to six times per day. Besides, most existing IM acupuncture programs require three times of consultation follow-up per week. We previously demonstrated that convenience of access is a key barrier of IM service implementation (6). Strategies to reduce the frequency of oral TCIM medication intake and integrate TCIM service delivery into the workflow of ConM would be important to enhance the service utilization and compliance.

### Using Add-On Design With Standard Care Comparator to Inform Integrative Practice

Most existing studies used standard care or placebo/sham acupuncture as comparator. While placebo minimizes various kinds of bias, it is not an ideal control in pragmatic trial design as patients are neither blinded nor receiving placebo in real-world practice (1). Furthermore, our focus group series shows that both patients and clinicians focus on the add-on effect of TCIM. The add-on effect would be difficult to assess if other active interventions are used as comparator.

N-of-1 design is advocated in pragmatic trial to evaluate programs with individualized intervention (45). TCIM, including CM, strongly emphasizes personalization with tailor-made treatment and each patient would be an ideal self-control (6). However, the assumption underpinning N-of-1 design is that the intervention would not have a long-term effect after cessation. This assumption is contradictory to the theory of many streams of TCIM which consider that TCIM can restore the balance of human constitution and therefore offers a long-term healing effect (6, 46). As the latent effect of TCIM is often a subject of interest, the wash-out period of N-of-1 trial needs to be long enough and should be justified by pilot studies.

### Implementation Challenges on Using TCIM Diagnosis as Inclusion Criteria

Five studies from our systematic review included TCIM-specific symptom-based diagnosis in the inclusion/exclusion criteria. Some streams of TCIM, for instance, CM, has a different epistemology compared to ConM, including disease stratification (6). CM defines disease predominantly according to phenotype. We previously demonstrated that add-on symptom-based diagnosis independently predicts renal progression among diabetic patients (47). Using standardized treatment across a study population with different CM-specific diagnosis is not personalized and contradictory to CM practice (6). As pragmatic trials are designed to reflect and inform real-world practice, CM-specific diagnosis is necessary in defining CM subgroups for intervention and assessment.

**Figure 5:**
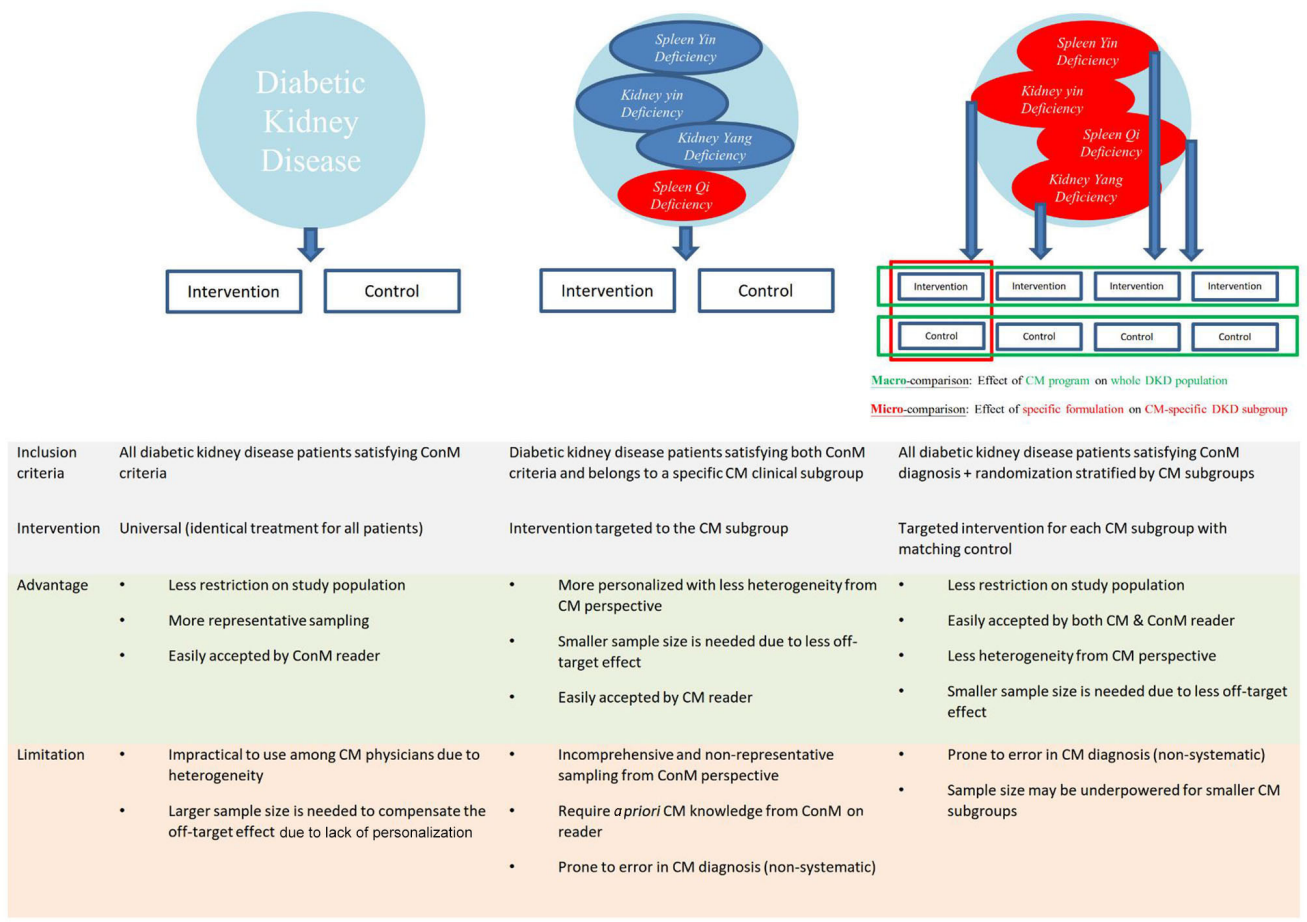
Schematic integration of diagnostic criteria of Chinese medicine and conventional medicine for diabetic kidney disease. Proposed integration of diagnostic criteria of Chinese medicine (CM) and conventional medicine (ConM) is used as illustration for other traditional, complementary, and integrative medicine (TCIM). Recruiting all diabetic kidney disease (DKD) patients with subsequent randomization stratified by CM diagnosis provides good representation of DKD population, reflect real-world practice of CM, and can be easily interpreted by both ConM and CM physicians. Macro-comparison by combining all subgroups evaluates the effectiveness of CM program to inform ConM physicians on necessary referral. Micro-comparison by subgroup analyses evaluates the effectiveness of each CM regimen for the corresponding subgroup to inform CM physicians on the personalized effect.

However, evidence generated from a specific subgroup of patients based on CM diagnosis may not be generalizable to the whole disease population (Figure 5) (48). For example, a formulation effective among diabetes patients that presented with *qi-yin deficiency* may not be effective among those without *qi-yin deficiency*, and therefore, the evidence has limited external validity to the whole diabetes population. As majority of ConM physicians are not trained in CM, evidence from trials that only recruited a subset of patients defined by symptoms could not inform ConM physicians' decision in referring patients for IM service.

To facilitate the implementation of evidence to IM service, we propose not to include TCIM-specific diagnosis in the inclusion/exclusion criteria of IM pragmatic trials to maximize the representation of the study population of interest (49). TCIM-specific diagnosis can be included as a stratification factor in randomization instead to generate TCIM-specific subgroups for analysis (Figure 5). By combining all subgroups which represents a whole disease population, the primary analysis evaluates the overall effectiveness of a TCIM service program that is executed according to TCIM real-world practice (49). The main analysis informs ConM physicians on whether to make necessary referral to IM service. Subgroup analysis stratified according to TCIM theory evaluates the effectiveness of different treatments given to each TCIM-specific subgroup. The subgroup analyses inform TCIM physicians the choice of modalities from a personalized perspective. This two-layer design maximizes the generalizability of evidence and translation to real-world practice for both ConM and TCIM physicians.

### Strategies to Maximize Reproducibility and Internal Validity in Pragmatic Trials

Although there are concerns over the trade-off between pragmatism and internal validity, our analysis showed that there is no positive correlation between risk of bias and pragmatism in existing study designs. Bias from randomization, allocation concealment, outcome assessment, and reporting in pragmatic trials can be controlled similarly to conventional trial designs (2). However, the intervention evaluated by pragmatic trials are often programs requiring flexibility, and the reproducibility is scrutinized (1). Although an unrestricted replicate of the real-world practice best produces evidence on effectiveness for implementation, the protocol may neither be applicable to nor reproducible in other clinical settings as high-quality standardized diagnostic instruments are lacking (50–52). For instance, the CM symptom-based diagnosis and personalized treatment in diabetes involves subjective professional judgment and likely differs between CM physicians. Although objective biomarkers may serve as alternative diagnostics, subjective symptom measures have been consistently demonstrated to correlate significantly with long-term clinical outcome independently (47, 53, 54) and has unique clinical value in patient-centered care (11).

To enhance the validity and reproducibility, symptom-based diagnosis and the corresponding variations in treatment should be pre-specified in a semi-individualized manner (49). Instead of diagnosing and treating patients purely by professional judgment that gives rise to unlimited combinations, patients can be divided by a predefined number of groups based on TCIM diagnosis with prespecified criteria. The treatment plan can be prespecified accordingly with clear instructions on adjustment. An alternative approach is to randomize or stratify the factor causing these variations, in most cases, the physician deciding the diagnosis and treatment. The potential confounding effect from different physicians can therefore be balanced between arms. However, a large cohort of subjects is needed for this method.

Non-uniform observation period is another commonly encountered challenge in pragmatic trial design. Most clinical trials would consider terminating subjects when serious adverse events develop due to clinical need and ethics concern, especially for patients under intervention in open-label design. As pragmatic trials often use standard care as control, subjects receiving standard care can be observed continuously without disturbing clinical management when serious adverse events develop. The imbalance in the length of observation between arms may confound outcome assessment especially for trials involving a long observation period and high incidence of serious adverse events, for instance, diabetes and CKD trials (55). A standardized termination criteria across arms upon developing serious adverse events can balance the observation length. Besides, using slope of change instead of absolute change in quantitative outcomes and incidence rate instead of incidence in count outcomes can also minimize the confounding from non-uniform follow-up.

### Quality of Reporting

Overall, the quality of reporting of the included studies is suboptimal, often with limited information for assessing the completeness of outcome reporting. The prospective registration of a trial and/or protocol publication with clearly prespecified outcome measurements before completion of a study can increase the transparency of outcome reporting. Also, the handling of missing values in the statistical analysis was also unclear. The use of less biased statistical methods in handling attrition (e.g., mixed regression model) with sensitivity analyses could enhance the internal validity of the results. Several studies have high risk of bias in outcome assessment as assessors were not blinded. Although pragmatic trials are often open-label among subjects and investigators, the blinding of the outcome assessor (e.g., by independent laboratory/assessor) is critical to reduce the potential observer bias in outcome assessment.

### Strengths and Limitations

This is the first focus group series to explore the specific expectation of the patients and physicians regarding IM diabetes and renal service, involving patients and family medicine, internal medicine, and CM physicians. A mixed-method approach was used in this study. The expectation of stakeholders was qualitatively explored to maximize the finding of mechanisms, and the *status quo* of clinical trial design was evaluated objectively and systematically with quantitative methods. This study has several limitations. As the focus group series focused on identifying detailed expectations on integrative Chinese-western medicine diabetes and CKD management, findings could be context specific (6). Nevertheless, CM is the mainstream of TCIM and most of the papers identified from the systematic review used CM as the intervention. Also, focus group interviews only delineate possible mechanisms of behavior. Further quantitative studies including surveys are needed to quantify the magnitude of the concerns and test the generalizability in other diseases. The priority of recommendations on study design could be assessed by further consensus methods and surveys involving an extended scope of stakeholders (e.g., caregiver) (56, 57). In the systematic review, the lack of detailed reporting on methodology is partly attributed to journal word limit, which impeded the accuracy of assessment. The correlation analysis between risk of bias and pragmatism is likely underpowered, although all IM pragmatic trials were included. The best estimate of correlation only reflects the association from best available evidence currently. Lastly, the assessment in systematic review only evaluates the quality of trial design through reporting and may not reflect the true quality of trial execution, especially for study protocols.

## Conclusion

Patients expected IM service to retard disease progression, stabilize concomitant drug dosage and reduce potential side-effects associated with conventional treatment, which were not reflected in existing study designs. Further pragmatic studies should consider more systematic and in-depth quantitative analyses of incident concomitant drug change and adverse events. Majority of studies either recruited a non-representative proportion of patients as TCIM diagnosis was used as inclusion criteria, or not reflecting the real-world practice of TCIM by completely dropping TCIM diagnosis. A two-layer design incorporating TCIM-specific symptom-based diagnosis as a stratification factor maximizes the generalizability of evidence and translation to real-world practice for both ConM and TCIM physicians.

## Data Availability Statement

The raw data supporting the conclusions of this article will be made available by the authors, without undue reservation.

## Ethics Statement

The studies involving human participants were reviewed and approved by The University of Hong Kong/Hospital Authority Hong Kong West Cluster Institutional Review Board and Hong Kong East Cluster Research Ethics Committee. The patients/participants provided their written informed consent to participate in this study.

## Author Contributions

KC, ST, and TL conceived the study. KC and PL collected the interview data and performed the script analysis. KC, CL, and GC coordinated the focus group interviews. KC extracted literature from electronic database. YL and LG screened and assessed the literature. KC and ST drafted the manuscript. All authors involved in the interpretation of data and manuscript revision.

## Conflict of Interest

The authors declare that the research was conducted in the absence of any commercial or financial relationships that could be construed as a potential conflict of interest.

## Publisher's Note

All claims expressed in this article are solely those of the authors and do not necessarily represent those of their affiliated organizations, or those of the publisher, the editors and the reviewers. Any product that may be evaluated in this article, or claim that may be made by its manufacturer, is not guaranteed or endorsed by the publisher.

## Abbreviations

TCIM: traditional, complementary and integrative medicine
CKD: chronic kidney disease
CM: Chinese medicine
DKD: diabetic kidney disease
GFR: glomerular filtration rate
IM: integrative medicine
ConM: conventional medicine.
