# Supplementary material for: PRAgmatic Clinical Trial Design of Integrative MediCinE (PRACTICE): A Focus Group Series and Systematic Review on Trials of Diabetes and Kidney Disease
Source: Front Med (Lausanne). 2021 Aug 27;8:668913. doi: 10.3389/fmed.2021.668913 (PMC8429603; doi:10.3389/fmed.2021.668913)
Supplement: Supplementary file 1 [file Data_Sheet_1.pdf]

PRAgmatic Clinical Trial design of Integrative mediCinE (PRACTICE):

a focus group series and systematic review on diabetes and kidney disease

Supplementary File

		Page
S1	Search strategy of the systematic review	2
S2	List of excluded studies of systematic review	6
S3	Sensitivity analysis of the correlation between risk of bias and pragmatism	22

Supplementary S1 Search strategy of the systematic review

EMBASE

1. (((pragmatic$ or naturalistic or real world or real life or unblinded or unmasked or cluster or step$ wedge$ or point of care or factorial or switchback or switch back or phase 4 or phase IV) adj10 (study or trial)) or (practical trial or effectiveness trial or ((cluster$ or communit$) adj2 randomi$))).tw. [72083]
2. (general practice$ or primary care or registry based or health record$ or medical record$ or EHR or EMR or administrative data or routinely collected data or (communit$ adj2 intervention$) or quality improvement or implementation or decision support or health service$ or health system$ or comparative effectiveness or CER or usual care or evidence based or practice guideline$ or (guideline$ adj1 recommend$) or knowledge translation or health technology assessment or HTA or cost effectiveness or process evaluation or economic evaluation or patient oriented).tw. [1227504]
3. randomized controlled trial.pt. or ((comparative effectiveness or randomi?ed) adj10 (trial or study)).ti. [228326]
4. (comment on or phase 1 or phase I or phase 2 or phase II or non-randomi?ed or quasi-randomi?ed or pseudo-randomi?ed).ti. or (clinical trial, phase I or clinical trial, phase II or systematic review or meta* n2 *analysis or meta-analysis or review or editorial).pt. [4813719]
5. pragmatic trial/ [873]
6. ((1 OR 2) AND (3 NOT 4) OR 5 [31228]
7. exp Animals/ NOT Humans/ [12984153]
8. 6 NOT 7 [15780]
9. ((1 and 3) NOT 4) OR 5 [13974]
10. 9 NOT 7 [6912]
11. ((($integrat$ or $alternat$ or $complement$ or $chinese$ or $herb$) adj3 $medicine$) or $acup$ or $qigong$).mp. [177344]
12. 8 AND 11 [346]
13. 10 AND 11 [124]
14. $diabet$ or DM or $mellitus$ [1621099]
15. $kidney$ or $renal$ or $nephro$ or CKD or $ESRD$ or $ERKD$ [1743126]
16. 14 OR 15 [3141935]
17. 12 AND 16 [61]
18. 13 AND 16 [19]

Cochrane Library

1. (((pragmat* OR naturalistic OR real world OR real life OR unblinded OR unmasked OR cluster OR step* wedge* OR point of care OR factorial OR switchback OR switch back OR phase 4 OR phase IV) n10 (study OR trial)) OR (practical trial OR effectiveness trial OR ((cluster* or communit*) n2 random*))).tw. [9400]
2. (general practice* OR primary care OR registry based OR health record* OR medical record* OR EHR OR EMR OR administrative data OR routinely collected data OR (communit* n2 interven*) OR quality improvement OR implementation OR decision support OR health service* OR health system* OR comparative effectiveness OR CER OR usual care OR evidence based OR practice guideline* OR (guideline* n1 recommend*) OR knowledge translation OR health technology assessment OR HTA OR cost effectiveness OR process evaluation OR economic evaluation OR patient oriented).tw. [9393]
3. randomi*ed controlled trial OR comparative effectiveness n10 (trial OR study) [988918]
4. comment on OR phase 1 OR phase I OR phase 2 OR phase II OR non-randomi*ed OR quasi-randomi*ed OR pseudo-randomi*ed OR clinical trial, phase I OR clinical trial, phase II OR systematic review OR meta* n2* analysis OR review OR editorial [1676575]
5. pragmatic clinical trial [8262]
6. ((#1 OR #2) AND (#3 NOT #4)) OR #5 [8262]
7. animal* NOT human* [3844]
8. #6 NOT #7 [8254]
9. ((#1 AND #3) NOT #4) OR #5 [8262]
10. (*integrat* or *alternat* or *complement* or *chinese* or herb*) n3 *medicine* OR *acup* OR *qigong [20031]
11. #8 AND #10 [343]
12. #9 NOT #7 [8254]
13. #10 AND #12 [343]
14. *diabet* or *DM* or *mellitus* [585591]
15. kidney* or renal* or nephro* or CKD* or ESRD* or ERKD* [92840]
16. #14 or #15 [632538]
17. #11 AND #16 [206]
18. #13 AND #16 [206]

PubMed

1. (((pragmat* OR naturalistic OR real world OR real life OR unblinded OR unmasked OR cluster OR step* wedge* OR point of care OR factorial OR switchback OR switch back OR phase 4 OR phase IV) n10 (study OR trial)) OR (practical trial OR effectiveness trial OR ((cluster* or communit*) n2 random*))).tw. [3816]
2. (general practice* OR primary care OR registry based OR health record* OR medical record* OR EHR OR EMR OR administrative data OR routinely collected data OR (communit* n2 interven*) OR quality improvement OR implementation OR decision support OR health service* OR health system* OR comparative effectiveness OR CER OR usual care OR evidence based OR practice guideline* OR (guideline* n1 recommend*) OR knowledge translation OR health technology assessment OR HTA OR cost effectiveness OR process evaluation OR economic evaluation OR patient oriented).tw. [25596]
3. randomi*ed controlled trial OR comparative effectiveness n10 (trial OR study)) [878554]
4. comment on OR phase 1 OR phase I OR phase 2 OR phase II OR non-randomi*ed OR quasi-randomi*ed OR pseudo-randomi*ed OR clinical trial, phase I OR clinical trial, phase II OR systematic review OR (meta* n2 *analysis) OR review OR editorial
5. pragmatic clinical trial [4972803]
6. animals NOT humans [4426682]
7. ((#1 OR #2) AND (#3 NOT #4)) OR #5 [4037]
8. #7 NOT #6 [4012]
9. ((#1 AND #3) NOT #4) OR #5 [3886]
10. #9 NOT #6 [3861]
11. ((*integrat* OR *alternat* OR *complement* OR *chinese* OR *herb*) n3 *medicine*) OR *acup* or *qigong* [36478]
12. #8 AND #11 [84]
13. #10 AND #11 [85]
14. kidney* or renal* or nephro* or CKD* or ESRD* or ERKD* [1,234,831]
15. *diabet* or DM or *mellitus* [817219]
16. #14 or #15 [1,929,249]
17. #12 AND #16 [9]
18. #13 AND #16 [9]

MedLine

1. (((pragmatic$ or naturalistic or real world or real life or unblinded or unmasked or cluster or step$ wedge$ or point of care or factorial or switchback or switch back or phase 4 or phase IV) adj10 (study or trial)) or (practical trial or effectiveness trial or ((cluster$ or communit$) adj2 randomi$))).tw. [38117]
2. (general practice$ or primary care or registry based or health record$ or medical record$ or EHR or EMR or administrative data or routinely collected data or (communit$ adj2 intervention$) or quality improvement or implementation or decision support or health service$ or health system$ or comparative effectiveness or CER or usual care or evidence based or practice guideline$ or (guideline$ adj1 recommend$) or knowledge translation or health technology assessment or HTA or cost effectiveness or process evaluation or economic evaluation or patient oriented).tw. [723120]
3. randomized controlled trial.pt. or ((comparative effectiveness or randomi?ed) adj10 (trial or study)).ti. [522607]
4. (comment on or phase 1 or phase I or phase 2 or phase II or non-randomi?ed or quasi-randomi?ed or pseudo-randomi?ed).ti. or (((clinical trial, phase I or clinical trial, phase II or systematic review or meta*).mp. adj2 *analysis/) or review.mp. or editorial.mp.) [3469516]
5. pragmatic clinical trial.pt. [1466]
6. ((1 or 2) and (3 not 4)) or 5 [41176]
7. exp Animals/ not Humans/ [4725507]
8. 6 not 7 [41028]
9. ((1 and 3) not 4) or 5 [14710]
10. 9 not 7 [14636]
11. ((($integrat$ or $alternative$ or $alternat$ or $complement$ or $chinese$ or $herb$) adj3 $medic$) or $acup$ or $qigong$).mp [94030]
12. 8 AND 11 [617]
13. 10 AND 11 [192]
14. $diabet$ or DM or $mellitus$ [743255]
15. $kidney$ or $renal$ or $nephro$ or CKD or $ESRD$ or $ERKD$ [1055798]
16. 14 OR 15 [1706017]
17. 12 AND 16 [37]
18. 13 AND 16 [13]

Supplementary S2 List of excluded studies of systematic review

ID	First Author	Title	Reason^
1	C. L. B. Dennis	Interventions (other than psychosocial, psychological and pharmacological) for treating postpartum depression	3
2	E. T. Faltinsen	Placebo, usual care and wait-list interventions for all mental health disorders	3
3	A. D. Y. Furlan	Acupuncture for (sub)acute non-specific low-back pain	3
4	G. G. Hilde	Physical therapy interventions for pelvic girdle pain (PGP) after pregnancy	3
5	V. S. Labonté	Algorithm-based pain management for people with dementia in nursing homes	3
6	M. S. N. Lee	Acupuncture for chronic neck pain	3
7	J. R. U. Lyttle	Antidepressants for osteoarthritis	3
8	R. F. Street	Participation of traditional, complementary and alternative health practitioners in conventional health systems in low- and middle-income countries	3
9	X. M. Zhu	Chinese medicine for immunological functions in women with breast cancer or a history of breast cancer	5
10	C. A. O. Anderson	Conservative management for postprostatectomy urinary incontinence	5
11	B. S. L. Buckley	Drugs for treatment of urinary retention after surgery in adults	5
12	J. A. N. K. Dorresteijn	Patient education for preventing diabetic foot ulceration	5
13	R. C. D. Hoogeveen	Complex interventions for preventing diabetic foot ulceration	5
14	A. G. Hróbjartsson	Placebo interventions for all clinical conditions	5
15	M. S. Imamura	Interventions for treating people with symptoms of bladder pain syndrome: a network meta-analysis	5
16	S. M. W. Smith	Interventions for improving outcomes in patients with multimorbidity in primary care and community settings	5
17	G. P. Virgili	Anti-vascular endothelial growth factor for diabetic macular oedema: a network meta-analysis	5
18	E. J. P. Bryan	Zuclopenthixol dihydrochloride for schizophrenia	4

19	N. D. Chuchu	Teledermatology for diagnosing skin cancer in adults	4
20	E. F. da Silva Freire Coutinho	Zuclopenthixol decanoate for schizophrenia and other serious mental illnesses	4
21	N. J. Ivers	Audit and feedback: effects on professional practice and healthcare outcomes	4
22	K. G. Jayakody	Zuclopenthixol acetate for acute schizophrenia and similar serious mental illnesses	4
23	E. F. S. B. Kaner	Effectiveness of brief alcohol interventions in primary care populations	4
24	Z. T. Munn	Rinse-free hand wash for reducing absenteeism among preschool and school children	4
25	N. D. Sturman	Methylphenidate for children and adolescents with autism spectrum disorder	4
26	Z. A. Alfirevic	Oral misoprostol for induction of labour	3
27	S. C. Allida	Pharmacological, psychological and non-invasive brain stimulation interventions for preventing depression after stroke	3
28	S. C. Allida	Pharmacological, psychological, and non-invasive brain stimulation interventions for treating depression after stroke	3
29	I. B. Arends	Interventions to facilitate return to work in adults with adjustment disorders	3
30	A. S. Atchabahian	Regional analgesia for improvement of long-term functional outcome after elective large joint replacement	3
31	A. L. Azarpazhooh	Xylitol for preventing acute otitis media in children up to 12 years of age	3
32	P. R. A. F. Baker	Interventions for preventing abuse in the elderly	3
33	E. L. O.-B. Ball	Aromatherapy for dementia	3
34	M. H. F. Bastos	Debriefing interventions for the prevention of psychological trauma in women following childbirth	3
35	P. K. Bower	Counselling for mental health and psychosocial problems in primary care	3
36	N. K. E. Bradford	Normal saline (0.9% sodium chloride) versus heparin intermittent flushing for the prevention of occlusion in long-term central venous catheters in infants and children	3
37	M. C. K. Brady	Speech and language therapy for aphasia following stroke	3
38	A. C. Brignell	Communication interventions for autism spectrum disorder in minimally verbal children	3

39	J. C. Broderick	Yoga versus non-standard care for schizophrenia	3
40	T. M. Brown	Interventions for preventing obesity in children	3
41	I. D. D. Cameron	Interventions for preventing falls in older people in care facilities and hospitals	3
42	D. K. L. W. Cheuk	Acupuncture for epilepsy	3
43	D. K. L. Y. Cheuk	Acupuncture for insomnia	3
44	E. R. B. Cluett	Immersion in water during labour and birth	3
45	J. A. S. David	Injected corticosteroids for treating plantar heel pain in adults	3
46	J. C. Z. Deare	Acupuncture for treating fibromyalgia	3
47	S. N. H. Every-Palmer	Pharmacological treatment for antipsychotic-related constipation	3
48	N. B. Fleeman	Care delivery and self-management strategies for children with epilepsy	3
49	A. L. Flower	Chinese herbal medicine for endometriosis	3
50	P. M. F. Fortin	Interventions for improving adherence to iron chelation therapy in people with sickle cell disease or thalassaemia	3
51	A. D. v. T. Furlan	Acupuncture and dry-needling for low back pain	3
52	A. C. M. Furmaniak	Exercise for women receiving adjuvant therapy for breast cancer	3
53	W. W. Gibson	Transcutaneous electrical nerve stimulation (TENS) for neuropathic pain in adults	3
54	W. W. Gibson	Transcutaneous electrical nerve stimulation (TENS) for chronic pain - an overview of Cochrane Reviews	3
55	A. J. K. Grande	Exercise versus no exercise for the occurrence, severity, and duration of acute respiratory infections	3
56	H. H. G. B. Handoll	Interventions for treating proximal humeral fractures in adults	3
57	H. H. G. E. Handoll	Rehabilitation for distal radial fractures in adults	3
58	P. J. C. Hay	Individual psychological therapy in the outpatient treatment of adults with anorexia nervosa	3
59	K. S. Head	Saline irrigation for allergic rhinitis	3

60	B. J. W. Hoare	Constraint-induced movement therapy in children with unilateral cerebral palsy	3
61	Z. N. Iheozor-Ejiofor	Negative pressure wound therapy for open traumatic wounds	3
62	S. S. James	Chelation for autism spectrum disorder (ASD)	3
63	T. D. M. Jefferson	Physical interventions to interrupt or reduce the spread of respiratory viruses	3
64	J. K. W. Jewer	Supplemental perioperative intravenous crystalloids for postoperative nausea and vomiting	3
65	K. K. Jeyashree	Smoking cessation interventions for pulmonary tuberculosis treatment outcomes	3
66	P. L. Jones	Oral non-steroidal anti-inflammatory drugs versus other oral analgesic agents for acute soft tissue injury	3
67	Z. Y. W. Ju	Acupuncture for neuropathic pain in adults	3
68	D. K. Kendrick	Exercise for reducing fear of falling in older people living in the community	3
69	T. S. Krisanaprakornkit	Meditation therapy for anxiety disorders	3
70	M. J. Lacey	Zuclopenthixol versus placebo for schizophrenia	3
71	L. B. Larun	Exercise therapy for chronic fatigue syndrome	3
72	T. A. G. Lawrie	Interventions to reduce acute and late adverse gastrointestinal effects of pelvic radiotherapy for primary pelvic cancers	3
73	S. F. W. Lensen	Individualised gonadotropin dose selection using markers of ovarian reserve for women undergoing in vitro fertilisation plus intracytoplasmic sperm injection (IVF/ICSI)	3
74	K. A. Linde	Acupuncture for the prevention of episodic migraine	3
75	N. K. Lindson	Smoking reduction interventions for smoking cessation	3
76	J. W. Liu	Treatment of epilepsy for people with Alzheimer's disease	3
77	E. A. K. Loveman	Parent-only interventions for childhood overweight or obesity in children aged 5 to 11 years	3
78	K. M. Machmutow	Comparative effectiveness of continuation and maintenance treatments for persistent depressive disorder in adults	3
79	E. C. Manheimer	Acupuncture for peripheral joint osteoarthritis	3

80	E. C. Manheimer	Acupuncture for treatment of irritable bowel syndrome	3
81	E. C. Manheimer	Acupuncture for hip osteoarthritis	3
82	T. J. V. E. Marin	Multidisciplinary biopsychosocial rehabilitation for subacute low back pain	3
83	A. L. C. P. Martimbianco	Transcutaneous electrical nerve stimulation (TENS) for chronic neck pain	3
84	J. C. McCleery	Pharmacotherapies for sleep disturbances in dementia	3
85	J. T. Mehrholz	Electromechanical-assisted training for walking after stroke	3
86	N. E. W. O'Connell	Interventions for treating pain and disability in adults with complex regional pain syndrome- an overview of systematic reviews	3
87	C. B. M. Oliveira	Epidural corticosteroid injections for lumbosacral radicular pain	3
88	M. J. M. W. Page	Splinting for carpal tunnel syndrome	3
89	C. A. J. Paley	Acupuncture for cancer pain in adults	3
90	N. K. Patel	Psychological, social and welfare interventions for psychological health and well-being of torture survivors	3
91	E. J. C. Peckham	Homeopathy for treatment of irritable bowel syndrome	3
92	I. A. Peytremann-Bridevaux	Chronic disease management programmes for adults with asthma	3
93	M. M. Pitkethly	Recordings or summaries of consultations for people with cancer	3
94	A. B. Pollock	Physical rehabilitation approaches for the recovery of function and mobility following stroke	3
95	M. G. Purgato	Psychological therapies for the treatment of mental disorders in low- and middle-income countries affected by humanitarian crises	3
96	A. C. Rankin	Interventions to improve the appropriate use of polypharmacy for older people	3
97	A. L. Schoonees	Ready-to-use therapeutic food (RUTF) for home-based nutritional rehabilitation of severe acute malnutrition in children from six months to five years of age	3
98	A. B. Shah	Transfusion of red blood cells stored for shorter versus longer duration for all conditions	3
99	E. S. Shepherd	Neonatal interventions for preventing cerebral palsy: an overview of Cochrane Systematic Reviews	3

100	C. F. Sherrington	Exercise for preventing falls in older people living in the community	3
101	K. H. Shinohara	Behavioural therapies versus other psychological therapies for depression	3
102	W. X. Siemens	Pharmacological interventions for pruritus in adult palliative care patients	3
103	S. A. L. Simpson	Oral or topical nasal steroids for hearing loss associated with otitis media with effusion in children	3
104	Y. S. Sinha	Auditory integration training and other sound therapies for autism spectrum disorders (ASD)	3
105	K. M. W. Smart	Physiotherapy for pain and disability in adults with complex regional pain syndrome (CRPS) types I and II	3
106	C. A. A. Smith	Acupuncture for depression	3
107	C. A. C. Smith	Aromatherapy for pain management in labour	3
108	C. A. L. Smith	Relaxation techniques for pain management in labour	3
109	C. A. L. Smith	Massage, reflexology and other manual methods for pain management in labour	3
110	L. A. B. Smith	Parenteral opioids for maternal pain management in labour	3
111	R. A. Syed	Pharmacological interventions for clozapine-induced hypersalivation	3
112	P. A. Tharyan	Electroconvulsive therapy for schizophrenia	3
113	J. S. Thorpe	Combination pharmacotherapy for the treatment of fibromyalgia in adults	3
114	W. H. R. van der Gaag	Non-steroidal anti-inflammatory drugs for acute low back pain	3
115	M. L. M. van Driel	Interventions to improve adherence to lipid-lowering medication	3
116	R. M. A. V. van Nispen	Low vision rehabilitation for better quality of life in visually impaired adults	3
117	A. P. S. P. Verhagen	Conservative treatments for whiplash	3
118	V. V. R. Vlassov	Low level laser therapy for treating tuberculosis	3
119	J. P. O. Vogel	Pharmacological and mechanical interventions for labour induction in outpatient settings	3

120	J. O. Wang	Sulpiride augmentation for schizophrenia	3
121	R. D. Wang	Interventions for unexplained infertility: a systematic review and network meta-analysis	3
122	J. A. Webster	Use of plastic adhesive drapes during surgery for preventing surgical site infection	3
123	E. J. E. Welsh	Interventions for bronchiectasis: an overview of Cochrane systematic reviews	3
124	A. R. R. White	Acupuncture and related interventions for smoking cessation	3
125	L. S. S. Wieland	Yoga treatment for chronic non-specific low back pain	3
126	K. R. Wilhelmus	Antiviral treatment and other therapeutic interventions for herpes simplex virus epithelial keratitis	3
127	M. L. Xu	Acupuncture for acute stroke	3
128	H. H. B. N. N. Yoo	Outpatient versus inpatient treatment for acute pulmonary embolism	3
129	C. H. Young	Home or foster home care versus institutional long-term care for functionally dependent older people	3
130	F. T. L. Zaina	Surgical versus non-surgical treatment for lumbar spinal stenosis	3
131	Y. P. Zhang	Acupuncture for uterine fibroids	3
132	E. N. C. Allen	Eliciting adverse effects data from participants in clinical trials	6
133	R. B. Langford	The WHO Health Promoting School framework for improving the health and well-being of students and their academic achievement	6
134	V. L. C. Luiza	Pharmaceutical policies: effects of cap and co-payment on rational use of medicines	6
135	H. E. G. Abdel-Aleem	Mobile clinics for women's and children's health	4
136	P. M. v. d. B. Archambault	Collaborative writing applications in healthcare: effects on professional practice and healthcare outcomes	4
137	M. S. Butler	Hospital nurse-staffing models and patient- and staff-related outcomes	4
138	J. E. H. Butterworth	Interventions for involving older patients with multi-morbidity in decision-making during primary care consultations	4
139	C. E. C. Chen	Walk-in clinics versus physician offices and emergency rooms for urgent care and chronic disease management	4

140	N. T. Chuchu	Smartphone applications for triaging adults with skin lesions that are suspicious for melanoma	4
141	A. J. E. Cross	Interventions for improving medication-taking ability and adherence in older adults prescribed multiple medications	4
142	S. V. Durao	Community-level interventions for improving access to food in low- and middle-income countries	4
143	G. T. Eamer	Comprehensive geriatric assessment for older people admitted to a surgical service	4
144	G. R. Flodgren	Interactive telemedicine: effects on professional practice and health care outcomes	4
145	C. C. Glenton	Barriers and facilitators to the implementation of lay health worker programmes to improve access to maternal and child health: a qualitative evidence synthesis	4
146	M. G. Gordon	Parent training programmes for managing infantile colic	4
147	D. J. M. Gould	Interventions to improve hand hygiene compliance in patient care	4
148	E. F. S. B. Kaner	Personalised digital interventions for reducing hazardous and harmful alcohol consumption in community-dwelling populations	4
149	M. v. d. B. Laurant	Nurses as substitutes for doctors in primary care	4
150	L. H. O'Doherty	Screening women for intimate partner violence in healthcare settings	4
151	A. W. Oyo-Ita	Interventions for improving coverage of childhood immunisation in low- and middle-income countries	4
152	T. G. Pantoja	Manually-generated reminders delivered on paper: effects on professional practice and patient outcomes	4
153	P. M. Posadzki	Automated telephone communication systems for preventive healthcare and management of long-term conditions	4
154	G. G. Weeks	Non-medical prescribing versus medical prescribing for acute and chronic disease management in primary and secondary care	4
155	J. B. Fabes	Pro-coagulant haemostatic factors for the prevention and treatment of bleeding in people without haemophilia	4
156	S. M. James	Omega-3 fatty acids supplementation for autism spectrum disorders (ASD)	4
157	M. W. Johansen	Prothrombin complex concentrate for reversal of vitamin K antagonist treatment in bleeding and non-bleeding patients	4
158	G. W. Norman	Dressings and topical agents for treating venous leg ulcers	4
159	C. B. S. Scallon	Flavonoids for treating venous leg ulcers	4
160	Nct	The Management of Diabetes in Everyday Life Program	4

162	J. Hughes	Ensuring model validity in a feasibility study of acupuncture to improve quality of life in cancer patients undergoing radiotherapy treatment	3
163	Actrn	Acupuncture or ear acupuncture for weight loss in Polycystic Ovary Syndrome	3
164	J. P. Bellmann-Strobl	The effectiveness of acupuncture and mindfulness-based stress reduction (MBSR) for patients with multiple sclerosis associated fatigue - A study protocol and its rationale for a randomized controlled trial	3
165	S. P. Blodt	Effectiveness of additional self-care acupressure for women with menstrual pain compared to usual care alone: a two-armed randomized pragmatic trial	3
166	Y. T. Z. Chan	Effectiveness and safety of acupuncture for poststroke dysphagia: study protocol for a pragmatic multicenter nonrandomized controlled trial	3
167	W. T. S. Chen	Evaluation on the effect of acupuncture on patients with sepsis-induced myopathy (ACU-SIM pilot study): a single center, propensity-score stratified, assessor-blinded, prospective pragmatic controlled trial	3
168	ChiCtr	Acupuncture for Cancer Pain: a pilot pragmatic randomised controlled trial	3
169	V. C. H. W. Chung	Electroacupuncture plus standard of care for managing refractory functional dyspepsia: protocol of a pragmatic trial with economic evaluation	3
170	V. C. W. Chung	Electroacupuncture plus on-demand gastrocaine for refractory functional dyspepsia: pragmatic randomized trial	3
171	M. M. S. Cohen	Acupuncture for analgesia in the emergency department: a multicentre, randomised, equivalence and non-inferiority trial	3
172	C. X. Ee	Acupuncture for menopausal hot flushes: a randomised sham-controlled trial	3
173	P. H. Fisher	A feasibility study of Acupuncture to improve quality of life and fatigue in cancer patients undergoing Radiotherapy Treatment (ART)	3
174	G. F. Georgoudis	The effect of myofascial release and microwave diathermy combined with acupuncture versus acupuncture therapy in tension-type headache patients: a pragmatic randomized controlled trial	3
175	Y. M. He	Acupuncture for cancer pain: protocol for a pilot pragmatic randomised controlled trial	3
176	R. S. M. Hinman	Efficacy of acupuncture for chronic knee pain: protocol for a randomised controlled trial using a Zelen design	3
177	S. P. Jang	Study protocol of a pragmatic, randomised controlled pilot trial: clinical effectiveness on smoking cessation of traditional and complementary medicine interventions, including acupuncture and aromatherapy, in combination with nicotine replacement therapy	3
178	S. K. Lee	Moxibustion for treating knee osteoarthritis: study protocol of a multicentre randomised controlled trial	3

179	I. S. Liodden	Acupuncture versus usual care for postoperative nausea and vomiting in children after tonsillectomy/adenoidectomy: a pragmatic, multicentre, double-blinded, randomised trial	3
180	E. A. B. Lown	Acupressure to reduce symptoms of depression and anxiety in children in treatment for a childhood cancer and recipients of a hematopoietic stem cell transplant	3
181	H. T. MacPherson	Alexander Technique Lessons, Acupuncture Sessions or usual care for patients with chronic neck pain (ATLAS): study protocol for a randomised controlled trial	3
182	H. T. MacPherson	Alexander Technique Lessons or Acupuncture Sessions for Persons With Chronic Neck Pain: a Randomized Trial	3
183	F. K. Martins	Factors influencing further acupuncture usage and a more positive outcome in patients with osteoarthritis of the knee and the hip: a 3-year follow-up of a randomized pragmatic trial	3
184	Nct	Auricular Acupuncture for Acute Pain	3
185	Nct	Acupuncture or Self-Acupuncture in Managing Cancer-Related Fatigue in Women Who Have Received Chemotherapy for Stage I, Stage II, or Stage IIIA Breast Cancer	3
186	Nct	Acupuncture for the Treatment of Phantom Limb Syndrome	3
187	Nct	A Study on Effects of Acupressure Among the Frail Elderly in the Community Dwellings	3
188	Nct	Resistant Starch, Epigallocatechin Gallate and Chlorogenic Acid for Body Weight Loss in Menopause	3
189	Nct	Antipruritic Effect of Acupuncture in Patients With Atopic Dermatitis	3
190	Nct	Pragmatic Research of Acupuncture and Counseling eXtended to Inpatient Services	3
191	J. H. Painovich	Acupuncture in the inpatient acute care setting: a pragmatic, randomized control trial	3
192	E. M. Peckham	Smoking cessation intervention for severe mental ill health trial (SCIMITAR): a pilot randomised control trial of the clinical effectiveness and cost-effectiveness of a bespoke smoking cessation service	3
193	S. M. Schroer	Acupuncture, or non-directive counselling versus usual care for the treatment of depression: a pilot study	3
194	G. X. L. Shi	Acupuncture for Vascular Dementia: a Pragmatic Randomized Clinical Trial	3
195	E. G. T. Trevelyan	Acupuncture for the treatment of phantom limb syndrome in lower limb amputees: a randomised controlled feasibility study	3

196	T. I. W. Usichenko	Acupuncture Reduces Pain and Autonomic Distress During Injection of Local Anesthetic in Children: a Pragmatic Crossover Investigation	3
197	J. R. Vas	Study protocol for a pragmatic randomised controlled trial in general practice investigating the effectiveness of acupuncture against migraine	3
198	J. S. Y. Wang	Acupuncture combined with tamsulosin hydrochloride sustained-release capsule in the treatment of chronic prostatitis/chronic pelvic pain syndrome: a study protocol for a randomized controlled trial	3
199	Y. G. Wang	Characteristics and obstetrics outcomes of different traditional Chinese medicine syndromes in women with polycystic ovary syndrome: a secondary analysis	3
200	D. G. T. B. Whitehurst	The cost-effectiveness of acupuncture as an adjunct to exercise-based physiotherapy for osteoarthritis of the knee	3
201	S. W. Blodt	Women's reasons for participation in a clinical trial for menstrual pain: a qualitative study	5
202	Actrn	Can an allied health and nursing expanded scope Treatment Access Pathway (TAP) improve health outcomes for people with persistent pain? A pragmatic randomised controlled trial	4
203	S. P. Blodt	Effectiveness of app-based self-acupressure for women with menstrual pain compared to usual care: a randomized pragmatic trial	4
205	R. M. B. Clifford	A randomised controlled trial of a pharmaceutical care programme in high-risk diabetic patients in an outpatient clinic	4
206	M. H. De Groot	Program active II: A comparative effectiveness trial to treat major depression in T2DM	4
215	X. P. Wang	How to explore the effectiveness of traditional chinese herbal medicine in a pragmatic randomized controlled trial? Experiences from treating diabetic kidney disease	5
217	N. E. L. Avis	A randomized, controlled pilot study of acupuncture treatment for menopausal hot flashes	3
218	J. P. Bellmann-Strobl	The effectiveness of acupuncture and mindfulness-based stress reduction (MBSR) for patients with multiple sclerosis associated fatigue - A study protocol and its rationale for a randomized controlled trial	3
219	H. C. Chen	Naoxuekang, Xinnaoshutong and Xuesaitong capsules for treating stroke: A protocol for a randomised controlled trial	3
220	B. G. R. Druss	Integrated medical care for patients with serious psychiatric illness: A randomized trial	3
221	B. G. V. E. Druss	Randomized trial of an integrated behavioral health home: The Health Outcomes Management and Evaluation (HOME) study	3

222	H. F.-O. Elden	Acupuncture as an adjunct to standard treatment for pelvic girdle pain in pregnant women: Randomised double-blinded controlled trial comparing acupuncture with non-penetrating sham acupuncture	3
223	H. P. Essex	An economic evaluation of Alexander Technique lessons or acupuncture sessions for patients with chronic neck pain: A randomized trial (ATLAS)	3
224	S. V. Hayhoe	Reports on a large, pragmatic, randomised trial: Acupuncture for chronic headache in primary care (n=401)	3
225	I. H. Heo	Electroacupuncture as a complement to usual care for patients with non-acute low back pain after back surgery: A pilot randomised controlled trial	3
226	K. B. Johansson	Subacromial corticosteroid injection or acupuncture with home exercises when treating patients with subacromial impingement in primary care - A randomized clinical trial	3
227	J. M. L. Johnstone	Rationale and design of an international randomized placebo-controlled trial of a 36-ingredient micronutrient supplement for children with ADHD and irritable mood: The Micronutrients for ADHD in Youth (MADDY) study	3
228	Z. F. Kamenov	Evaluation of the efficacy and safety of Tribulus terrestris in male sexual dysfunction-A prospective, randomized, double-blind, placebo-controlled clinical trial	3
229	E. K. Kim	Effectiveness and Safety of Polydioxanone Thread-Embedding Acupuncture as an Adjunctive Therapy for Patients with Chronic Nonspecific Neck Pain: A Randomized Controlled Trial	3
230	Y. K. Y. Kim	Antipruritic Effect of Acupuncture in Patients with Atopic Dermatitis: Feasibility Study Protocol for a Randomised, Sham-Controlled Trial	3
231	F. M. L. Kovacs	Effectiveness and cost-effectiveness analysis of neuroreflexotherapy for subacute and chronic low back pain in routine general practice: A cluster randomized, controlled trial	3
232	L. F. Lai	Standardised versus individualised multiherb Chinese herbal medicine for oligomenorrhoea and amenorrhoea in polycystic ovary syndrome: A randomised feasibility and pilot study in the UK	3
233	W. L. Y. Lam	Combined electroacupuncture and auricular acupuncture for postoperative pain after abdominal surgery for gynecological diseases: Study protocol for a randomized controlled trial	3
234	S. H. S. Lee	Safety, effectiveness, and economic evaluation of an herbal medicine, Ukgansangajinpibanha granule, in children with autism spectrum disorder: A study protocol for a prospective, multicenter, randomized, double-blinded, placebo-controlled, parallel-group clinical trial	3
235	J. K. Leem	Efficacy and safety of thread embedding acupuncture combined with conventional acupuncture for chronic low back pain a study protocol for a randomized, controlled, assessor-blinded, multicenter clinical trial	3

236	M. P. Miladinia	The comparison of the effect of two complementary medicine methods (Music therapy and massage therapy) on postoperative acute pain after abdominal surgery: A randomized clinical trial study	3
237	D. P. Pach	Effectiveness and Cost-Effectiveness of Tuina for Chronic Neck Pain: A Randomized Controlled Trial Comparing Tuina with a No-Intervention Waiting List	3
238	S. L. T. Prady	The natural history of back pain after a randomised controlled trial of acupuncture vs usual care - Long term outcomes	3
239	T. K. B. Selfe	Effects of noninvasive interactive neurostimulation on symptoms of osteoarthritis of the knee: A randomized, sham-controlled pilot study	3
240	B. C. C. Shin	A multi-center, randomized controlled clinical trial, cost-effectiveness and qualitative research of electroacupuncture with usual care for patients with non-acute pain after back surgery: Study protocol for a randomized controlled trial	3
241	H. R. P. Shin	Chuna manual therapy combined with acupuncture and cupping for frozen shoulder (adhesive capsulitis): multicenter, randomized, patient-assessor blind, clinical trial	3
242	S. J. Shin	Effectiveness, safety, and economic evaluation of adjuvant moxibustion therapy for aromatase inhibitor-induced arthralgia of postmenopausal breast cancer stage i to III patients: Study protocol for a prospective, randomized, assessor-blind, usual-care controlled, parallel-group, pilot clinical trial	3
243	S. K. Shin	A Herbal Medicine, Gongjindan, in Subjects with Chronic Dizziness (GOODNESS Study): Study Protocol for a Prospective, Multicenter, Randomized, Double-Blind, Placebo-Controlled, Parallel-Group, Clinical Trial for Effectiveness, Safety, and Cost-Effectiveness	3
244	L. C. Shinto	A randomized pilot study of naturopathic medicine in multiple sclerosis	3
245	J. D. Thomas	Treatment of scabies using a tea tree oil-based gel formulation in Australian Aboriginal children: Protocol for a randomised controlled trial	3
246	J. A. Vas	Randomised controlled study in the primary healthcare sector to investigate the effectiveness and safety of auriculotherapy for the treatment of uncomplicated chronic rachialgia: A study protocol	3
247	J. C. Vas	Effect of ear acupuncture on pregnancy-related pain in the lower back and posterior pelvic girdle: A multicenter randomized clinical trial	3
248	J. M. Vas	Acupuncture as a complementary therapy to the pharmacological treatment of osteoarthritis of the knee: Randomised controlled trial	3
249	J. M. Vas	Effectiveness of acupuncture, special dressings and simple, low-adherence dressings for healing venous leg ulcers in primary healthcare: Study protocol for a cluster-randomized open-labeled trial	3

250	J. P.-M. Vas	Efficacy and safety of acupuncture for the treatment of non-specific acute low back pain: A randomised controlled multicentre trial protocol [ISRCTN65814467]	3
251	J. R. Vas	Study protocol for a pragmatic randomised controlled trial in general practice investigating the effectiveness of acupuncture against migraine	3
252	D. G. T. L. Whitehurst	A brief pain management program compared with physical therapy for low back pain: Results from an economic analysis alongside a randomized clinical trial	3
253	C. M. J. Witt	Pragmatic randomized trial evaluating the clinical and economic effectiveness of acupuncture for chronic low back pain	3
254	C. M. R. Witt	Acupuncture in patients with dysmenorrhea: a randomized study on clinical effectiveness and cost-effectiveness in usual care	3
255	D. V. Wonderling	Cost effectiveness analysis of a randomised trial of acupuncture for chronic headache in primary care	3
256	J. B. Woodman	Self-efficacy and self-care-related outcomes following Alexander Technique lessons for people with chronic neck pain in the ATLAS randomised, controlled trial	3
257	X. K. S.-V. Wu	Effect of acupuncture and clomiphene in Chinese women with polycystic ovary syndrome: A randomized clinical trial	3
258	J. L. Zhou	Cost-effectiveness of jingshu granules compared to placebo for the treatment of patients with cervical radiculopathy in China: A decision-tree model based on randomized controlled trial	3
259	P. D. Zou	Dietary approach to stop hypertension with sodium reduction for Chinese Canadians (Dashna-CC): A pilot randomized controlled trial	3
260	L. M. P.-L. Azogil-Lopez	DETELPROG study. Effectiveness of a new model of scheduled telephone referral from primary care to internal medicine. a randomised controlled study	4
261	K. L. N. Bennell	Effectiveness of an internet-delivered exercise and pain-coping skills training intervention for persons with chronic knee pain: A randomized trial	4
262	H. L. Chen	Comparative evaluation of novel screening strategies for colorectal cancer screening in China (TARGET-C): A study protocol for a multicentre randomised controlled trial	4
263	C. F. Strassner	Holistic care program for elderly patients to integrate spiritual needs, social activity, and self-care into disease management in primary care (HoPES3): Study protocol for a cluster-randomized trial	4
264	H. V. Motamed	Efficacy evaluation of nebulized magnesium, as an additional complementary treatment, in clinical and peak flow metric improvements of acute asthma attack: A randomized double-blinded clinical trial	4

266	M. S. de Groot	Program ACTIVE II: Outcomes From a Randomized, Multistate Community-Based Depression Treatment for Rural and Urban Adults With Type 2 Diabetes	4
269	J. L. Huo	Stationary Treatment Compared with Individualized Chinese Medicine for Type 2 Diabetes Patients with Microvascular Complications: Study Protocol for a Randomized Controlled Trial	8
270	D. H. Jin	Chinese herbal medicine TangBi Formula treatment of patients with type 2 diabetic distal symmetric polyneuropathy disease: study protocol for a randomized controlled trial	8
273	Y. Z. Liu	The clinical effect of traditional chinese medicine on middle-aged women with Interstitial Cystitis: Protocol for a randomized controlled trial	3
275	R. C. Remli	Use of complementary medicine amongst diabetic patients in a public primary care clinic in Ipoh	5
276	D. L. Rosenberg	Integrated medical care management and behavioral risk factor reduction for multicondition patients: behavioral outcomes of the TEAMcare trial	4
278	M. A. Vray	Randomized study of glibenclamide versus traditional Chinese treatment in type 2 diabetic patients. Chinese-French Scientific Committee for the Study of Diabetes	1
281	M. W. Wang	Effects of traditional Chinese herbal medicine in patients with diabetic kidney disease: study protocol for a randomized controlled trial	8
282	E. K. A. Borud	The acupuncture treatment for postmenopausal hot flushes (Acuflash) study: traditional Chinese medicine diagnoses and acupuncture points used, and their relation to the treatment response	3
283	L. A. v. Z. Donnelly	Robust association of the LPA locus with low-density lipoprotein cholesterol lowering response to statin treatment in a meta-analysis of 30 467 individuals from both randomized control trials and observational studies and association with coronary artery disease outcome during statin treatment	3
284	A. L. Flower	A feasibility study exploring the role of Chinese herbal medicine in the treatment of endometriosis	3
285	J.-H. J. Kim	Auricular acupuncture for prehypertension and stage 1 hypertension: study protocol for a pilot multicentre randomised controlled trial	3
286	H. T. MacPherson	Acupuncture for low back pain: traditional diagnosis and treatment of 148 patients in a clinical trial	3
287	R. K. Vedanthan	Bridging Income Generation with Group Integrated Care for cardiovascular risk reduction: Rationale and design of the BIGPIC study	3
288	Y.-f. Q. Wang	[Assessment of external methods of traditional Chinese medicine in patients with chronic ulcer of the lower extremities: study protocol of a multicenter, randomized, parallel-group, prospective trial]	3
289	Q. Z. Yuxi	Effects of Xuebijing Injection for Patients With Sepsis-induced Acute Kidney Injury After Wenchuan Earthquake	3

290	P. C. W. Morin	Record media used by primary care providers in medically underserved regions of upstate New York was not pivotal to clinical result in the Informatics for Diabetes Education and Telemedicine (IDEATel) project	4
291	D. G. C.-G. Morrow	An EMR-based tool to support collaborative planning for medication use among adults with diabetes: design of a multi-site randomized control trial	4
292	H. W. S. Rodbard	Use of an automated decision support tool optimizes clinicians' ability to interpret and appropriately respond to structured self-monitoring of blood glucose data	4
293	H. P. F. Rodriguez	The impact of integrating medical assistants and community health workers on diabetes care management in community health centers	4
294	C. F. Strasner	Holistic care program for elderly patients to integrate spiritual needs, social activity, and self-care into disease management in primary care (HoPES3): study protocol for a cluster-randomized trial	4
299	S. L. C. Tsay	Acupressure and Transcutaneous Electrical Acupoint Stimulation in improving fatigue, sleep quality and depression in hemodialysis patients	1
300	S. L. R. Tsay	Acupoints massage in improving the quality of sleep and quality of life in patients with end-stage renal disease	8
302	R. G. d. S.-C. Costa-Cavalcanti	Effect of Auriculotherapy on the Plasma Concentration of Biomarkers in Individuals with Knee Osteoarthritis	3
303	M. L. C. Yeh	A randomized controlled trial of auricular acupressure in heart rate variability and quality of life for hypertension	3

^1. full-text irretrievable, 2. language untranslatable, 3. not diabetes or kidney disease related, 4. not integrative medicine related, 5. not declared as pragmatic trial, 6. non-human study, 8. duplicate

Supplementary S3 Sensitivity analysis of the correlation between risk of bias and pragmatism

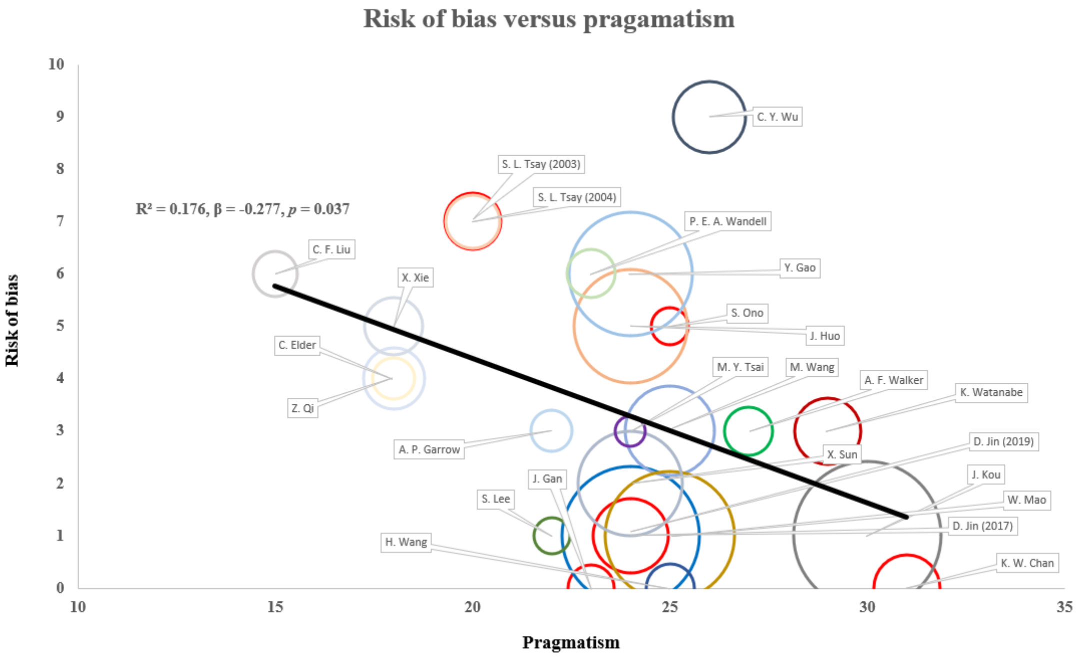

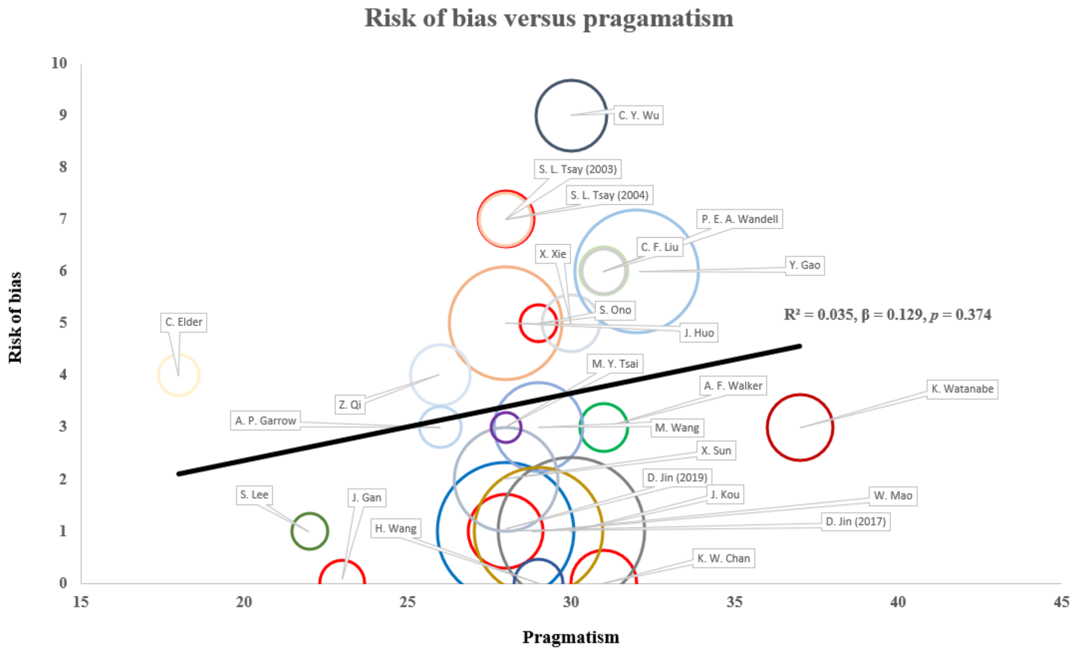

The risk of bias assessed based on Cochrane tool and pragmatism assessed based on PRECIS-2 are presented. Low, unknown and high risk in each domain scored 0, 1 and 2 in assessing the overall risk of bias of each study. Lower total score represents low risk of bias in reported study design. A domain scored 1 or 5 for being least or most pragmatic, respectively, according to the PRECIS-2 tool. Undetermined domain was replaced by 1 (lowest, above) and 5 (below). There is no statistically significant correlation between risk of bias and pragmatism in the multivariable regression models adjusting publication year and sample size. Summary statistics of univariable regression model is shown.
